# Therapy-induced senescence in breast cancer and cardiovascular disease: a double-edged biological process

**DOI:** 10.3389/fcvm.2026.1951892

**Published:** 2026-09-29

**Authors:** Eugenia Belcastro, Giuseppe N. Fanelli, Maria Luisa Schiavone, Federico Di Cocco, Chiara Cateni, Antonio G. Naccarato, Cristian Scatena, Valerie B. Schini-Kerth

**Affiliations:** 1 Department of Translational Research and New Technologies in Medicine and Surgery, University of Pisa, Pisa, Italy; 2 First Division of Pathology, Department of Translational Research and New Technologies in Medicine and Surgery, University of Pisa, Pisa, Italy; 3 Department of Oncology, Pisa University Hospital, Pisa, Italy; 4 UR 3074 Translational Cardiovascular Medicine, Biomedicine Research Center of Strasbourg, University of Strasbourg, Strasbourg, France

**Keywords:** breast cancer, cardio-oncology, cardiovascular dysfunction, inflammaging, senescence-targeting strategies, therapy-induced senescence

## Abstract

Therapy-induced senescence (TIS) has emerged as a central biological response to anticancer therapies, extending beyond tumor suppression to influence long-term tissue homeostasis, cardiovascular (CV) remodeling, and accelerated biological aging. In breast cancer (BC), chemotherapy, radiotherapy, targeted therapies, and emerging immune-activating treatments can induce senescence not only in malignant cells, where it may initially contribute to tumor growth arrest and immune-mediated clearance, but also in non-malignant CV tissues. Persistent accumulation of senescent cells and sustained release of the senescence-associated secretory phenotype (SASP) represent a double-edged sword that may promote chronic inflammation and endothelial dysfunction while contributing to the exacerbation of multifactorial CV outcomes, including myocardial fibrosis, vascular stiffness, and progressive CV dysfunction. Importantly, these outcomes cannot be attributed to TIS alone, as direct therapy-related injury and pre-existing CV risk factors may also contribute. Increasing evidence suggests that TIS-associated mechanisms — including persistent DNA damage response activation, p53/p21- and p16^INK4a^-mediated cell-cycle arrest, NF-*κ*B-driven inflammatory signaling, mitochondrial dysfunction, oxidative stress, and metabolic rewiring — overlap with pathways involved in CV toxicity and systemic tissue remodeling. Through chronic inflammatory and immune-vascular crosstalk, TIS may contribute to a broader phenotype of inflammaging and therapy-associated premature biological aging. Emerging senescence-targeting strategies, including senolytic and senomorphic therapies, represent promising approaches to mitigate long-term CV injury while preserving antitumor efficacy, although their clinical benefit and safety remain to be fully established. This review summarizes current knowledge regarding the dual and systemic role of TIS in BC and CV disease, considering TIS as a candidate, non-exclusive mediator acting alongside direct therapy-related injury, and discusses its translational implications for integrated precision cardio-oncology strategies.

## 1 Introduction

### 1.1 Therapy-induced senescence as a shared biological response linking antitumor efficacy and cardiovascular toxicity

Advances in breast cancer (BC) treatment have substantially improved disease control and long-term survival over recent decades. The integration of anthracyclines, taxanes, radio-, endocrine, HER2-targeted therapies and, more recently, CDK4/6 inhibitors and other targeted approaches has transformed the clinical trajectory of many patients with BC (1–3). Consequently, BC is now increasingly characterized by a large and expanding population of long-term survivors exposed to multimodal anticancer treatments. However, this therapeutic success has also shifted clinical attention toward late treatment-related toxicities and survivorship-associated comorbidities. Among these, cardiovascular disease has emerged as a major determinant of morbidity, quality of life impairment, and non-cancer-related mortality among BC survivors (1, 2, 4). This growing intersection between oncology and cardiovascular medicine has fueled the development of cardio-oncology, a field aimed not only at preventing, detecting, and managing overt cardiotoxicity but also at understanding the biological mechanisms through which life-saving anticancer therapies may inadvertently promote long-term cardiovascular dysfunction.

Among the biological processes increasingly implicated in both tumor response and systemic toxicity, therapy-induced senescence (TIS) has emerged as a potential shared mechanism linking anticancer efficacy with long-term tissue remodeling, cardiovascular dysfunction and accelerated cardiovascular aging (1, 3, 5). Although anthracyclines remain the best-characterized inducers of TIS, accumulating evidence indicates that several other anticancer modalities — including radiotherapy, HER2-targeted therapies, endocrine therapies, CDK4/6 inhibitors, and immune-modulating therapies — may also activate senescence-associated programs, albeit through partially distinct molecular mechanisms and tissue-specific stress responses. Taken together, these features make BC a particularly informative clinical model for investigating the intersection between therapy-induced senescence and cardiovascular disease. The prolonged and sequential use of multimodal treatments, encompassing a complex spectrum of cardiovascular risks extensively addressed in current cardio-oncology guidelines (6), combined with the predominantly female patient population, the influence of menopausal and hormonal status, and the growing number of long-term survivors, creates a distinctive clinical context in which delayed cardiovascular effects may emerge years or decades after successful cancer treatment. BC therefore provides a particularly relevant model for examining how initially beneficial or adaptive senescence responses may evolve into persistent systemic programs contributing to both tumor remodeling and long-term cardiovascular vulnerability. Throughout this review, evidence directly derived from breast cancer models is distinguished from mechanistic findings extrapolated from other tumor types or non-oncological cardiovascular settings, and the corresponding knowledge gaps are explicitly acknowledged.

Cellular senescence is classically characterized by a stable and long-lasting state of cell-cycle arrest triggered by a wide range of cellular stressors, including telomere attrition, oncogenic activation, oxidative stress, mitochondrial dysfunction and DNA damage (7–10). Initially described by Hayflick and Moorhead as a phenomenon associated with the progressive loss of proliferative capacity in normal human cells (11), senescence is now recognized as a complex and dynamic biological program involved not only in tumor suppression, but also in tissue remodeling, aging and chronic disease development (9, 12, 13).

Senescent cells remain metabolically active and exhibit characteristic molecular and phenotypic features, including persistent activation of the DNA damage response (DDR), increased expression of cyclin-dependent kinase inhibitors such as p21^CIP1 and p16^INK4a, chromatin remodeling, mitochondrial dysfunction, and secretion of a complex pro-inflammatory secretome collectively referred to as the senescence-associated secretory phenotype (SASP) (7, 9, 14, 15). Beyond cell-cycle arrest, senescent cells actively reshape their surrounding microenvironment through chronic inflammatory and metabolic signaling, thereby influencing tissue homeostasis and intercellular communication (8).

In the context of cancer, cellular senescence represents a major biological response to anticancer therapies. TIS is primarily driven by treatment-related genotoxic and oxidative stress and was initially considered a favorable therapeutic outcome because of its ability to induce stable proliferative arrest and facilitate immune-mediated clearance of damaged tumor cells (8, 16, 17). However, accumulating evidence suggests that senescence exerts highly context-dependent effects in cancer biology, functioning both as a tumor-suppressive and tumor-promoting process (9, 18). While senescence may initially act as a critical tumor-suppressive mechanism by limiting the proliferation of damaged cells through stable cell-cycle arrest (9, 18), persistent senescent cells may also promote tumor progression through SASP-mediated remodeling of the tumor microenvironment (TME) (13, 18), inflammation, epithelial–mesenchymal transition (EMT), immune escape, therapy resistance, and disease recurrence (8, 18–20). Importantly, senescent tumor cells may retain significant biological plasticity and, under specific conditions, escape from the senescent state, reacquiring proliferative and stem-like features associated with tumor progression and recurrence (8, 21, 22). Therefore, TIS is increasingly recognized not merely as a passive byproduct of anticancer treatment, but rather as a complex and dynamic process whose double-edged nature profoundly influences both tumor biology and systemic tissue homeostasis (8, 21).

Crucially, the senescence burden induced by anticancer therapies is not restricted to malignant tissues. Systemic anticancer therapies act as potent stressors capable of inducing premature aging phenotypes in non-malignant tissues, including the cardiovascular system. Cardiomyocytes, endothelial cells (ECs) — which appear to be among the earliest cardiovascular targets of therapy-induced stress, particularly within the arterial wall (23, 24) — fibroblasts, vascular smooth muscle cells, and immune cells may all acquire senescence-associated features after exposure to anticancer therapies, through mechanisms involving persistent DNA damage, oxidative stress, mitochondrial dysfunction, inflammatory activation, and impaired stress adaptation (1, 5, 25). The progressive accumulation of senescent cells within the heart and vasculature may contribute to endothelial dysfunction, impaired vascular repair, myocardial fibrosis, vascular stiffness, and chronic cardiovascular remodeling, thereby increasing long-term cardiovascular risk years after treatment completion, reinforcing the concept that the heart may represent an unintended victim of TIS (1, 2). Consistent with this view, growing evidence suggests that many senescence-associated mechanisms activated within tumors are also engaged in non-malignant tissues exposed to anticancer therapies, particularly within the cardiovascular system (5, 26). Experimental studies have demonstrated that oxidative stress-driven endothelial senescence is tightly associated with vascular dysfunction and premature vascular aging, further supporting the role of senescence-associated pathways in cardiovascular disease progression (27). The resulting low-grade chronic inflammatory milieu, commonly referred to as *inflammaging*, may further amplify tissue dysfunction and contribute to the progression of both cancer- and aging-related cardiovascular complications (26). In this context, therapy-induced senescence emerges as a shared biological axis linking antitumor efficacy with long-term cardiovascular vulnerability and therapy-associated premature aging (Figure 1).

**Figure 1:**
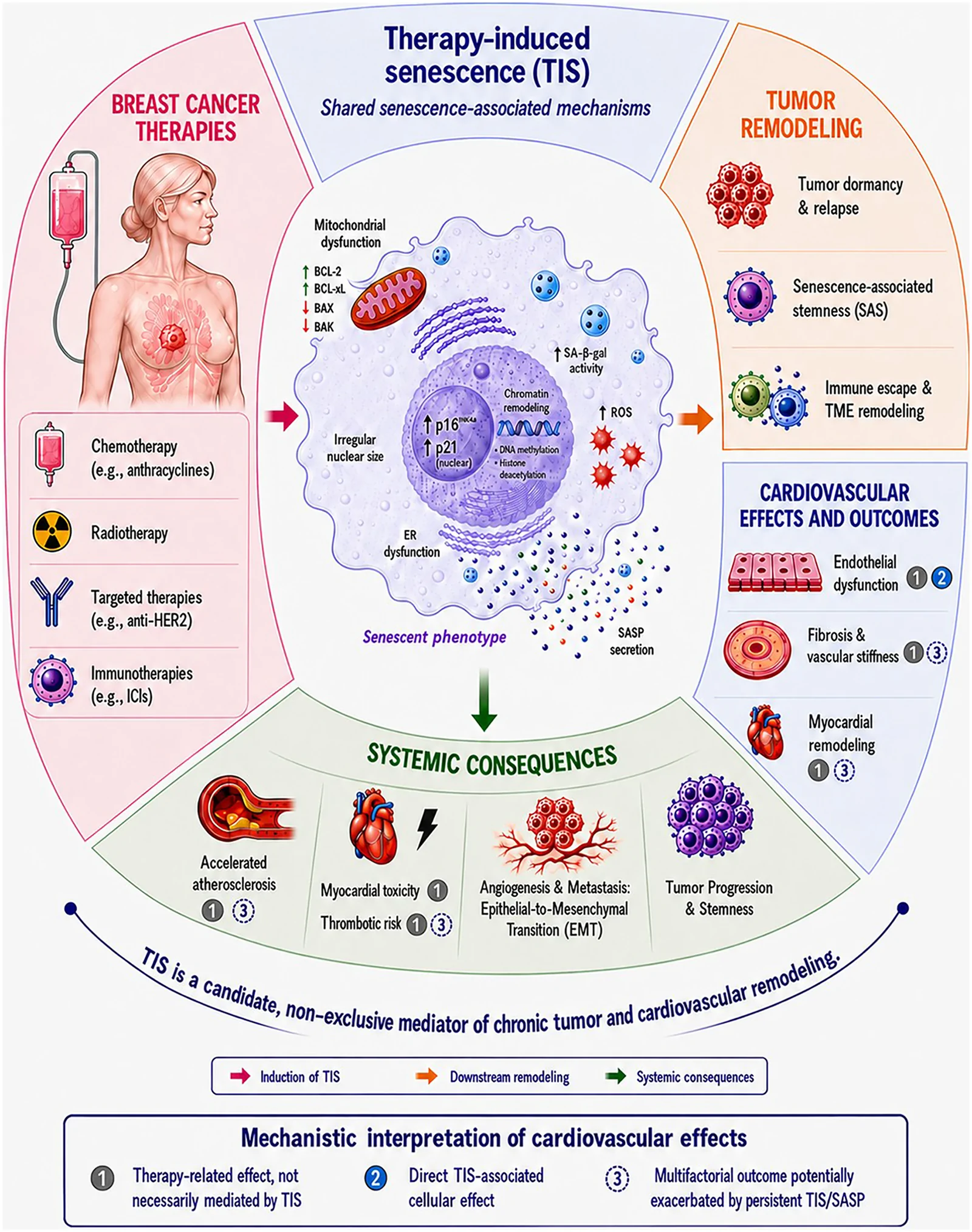
Therapy-induced senescence as a candidate biological bridge linking breast cancer treatment, cardiovascular remodeling, and accelerated aging. Breast cancer therapies, including chemotherapy, radiotherapy, targeted therapies, and immunotherapies, induce therapy-induced senescence (TIS) in both tumor and cardiovascular tissues. TIS is characterized by shared senescence-associated mechanisms, including mitochondrial dysfunction, chromatin remodeling, oxidative stress, apoptosis resistance, increased p16^INK4a^/p21^CIP1^ expression, and senescence-associated secretory phenotype (SASP) production. Persistent senescence may promote tumor remodeling through tumor dormancy and relapse, senescence-associated stemness (SAS), and immune escape associated with tumor microenvironment (TME) remodeling. Cardiovascular effects, characterized by endothelial dysfunction, fibrosis and vascular stiffness, and myocardial remodeling, are classified according to their mechanistic relationship with TIS; these categories are not mutually exclusive, as the same therapeutic insult may simultaneously induce direct cellular injury and promote a senescent phenotype. Local tumor and cardiovascular alterations may contribute to systemic consequences, including accelerated atherosclerosis, myocardial toxicity, thrombotic risk, angiogenesis and epithelial-to-mesenchymal transition (EMT), and tumor progression. Collectively, TIS represents a candidate, non-exclusive mediator of chronic tumor and cardiovascular remodeling, acting alongside direct therapy-related injury. Arrows indicate induction of TIS (pink), downstream tissue remodeling (orange) and systemic consequences (green). FigureLabs Plus (v4.1), an AI-powered scientific illustration platform, was used to assist with the generation of the graphical elements of this figure. The scientific content and graphical design were verified, reviewed, and finalized by the authors. Created with FigureLabs (https://figurelabs.ai).

In this review, we discuss the shared molecular and biological mechanisms through which TIS contributes to both BC progression and cardiovascular impairment, with particular focus on on SASP-driven inflammation, *inflammaging*, immune–vascular crosstalk, and senescence-associated remodeling. We further explore the emerging therapeutic potential of senescence-targeting strategies in cardio-oncology, including senotherapies aimed at modulating or eliminating senescent cells, as a possible approach to mitigate long-term cardiovascular dysfunction without compromising the antitumor efficacy of cancer treatment (1, 28). Understanding the dual and systemic role of TIS may provide novel insights into the pathophysiology of cancer therapy-related cardiovascular complications and support the development of integrated precision cardio-oncology strategies aimed at preserving both oncologic efficacy and cardiovascular health.

## 2 Molecular mechanisms of therapy-induced senescence

At the molecular level, TIS is orchestrated by a highly interconnected network of persistent DNA damage signaling, cell-cycle checkpoint activation, inflammatory activation, mitochondrial dysfunction, and metabolic rewiring. Although initially characterized as a tumor-suppressive response, these pathways are now recognized as conserved stress-response programs operating across both malignant and non-malignant tissues exposed to anticancer therapies. Importantly, the molecular pathways sustaining TIS are not restricted to TME, but they may be similarly activated within cardiovascular tissues, where they contribute to endothelial dysfunction, myocardial remodeling, vascular aging, fibrosis, and chronic inflammation (Figure 2).

**Figure 2:**
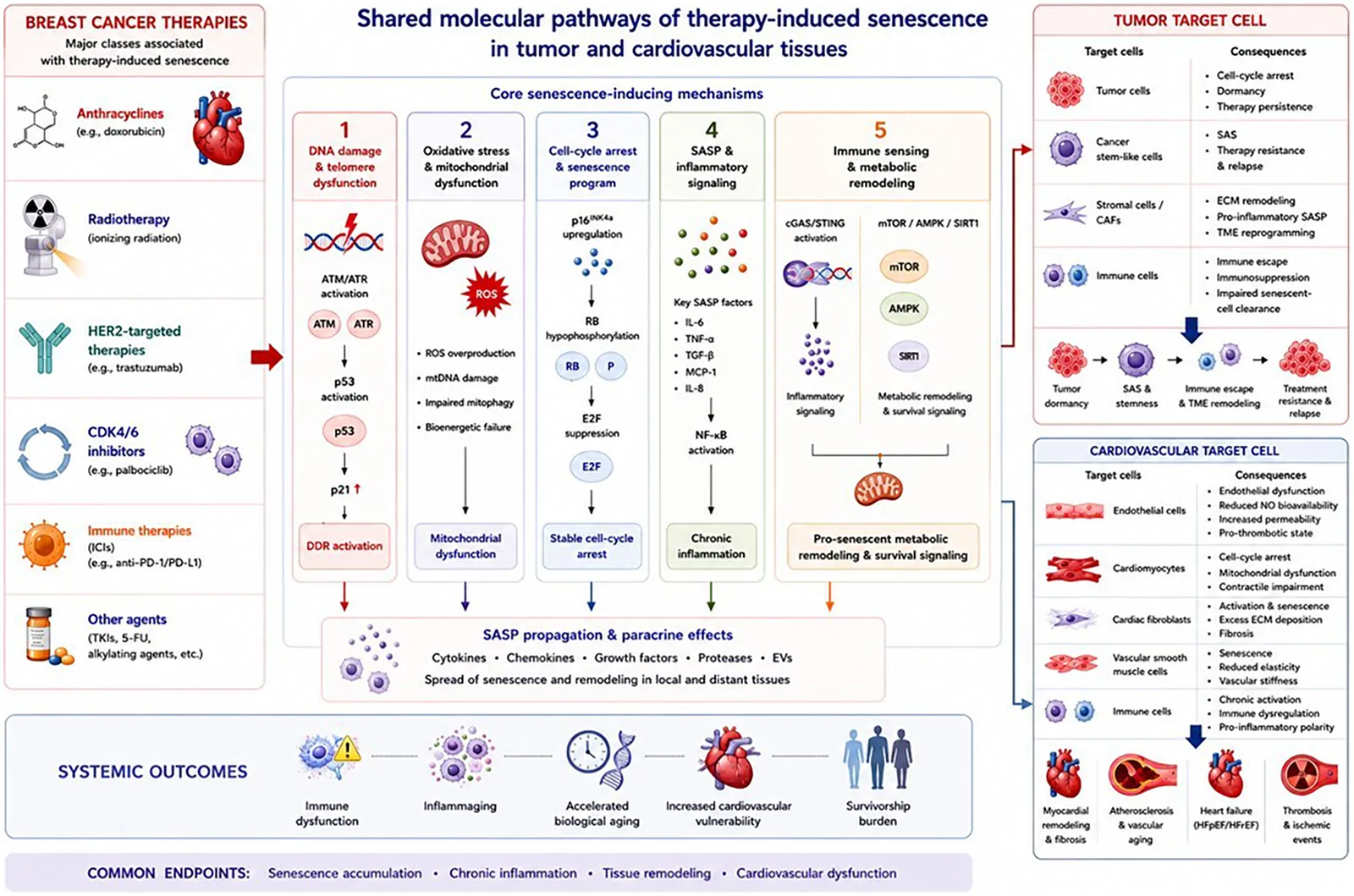
Shared molecular mechanisms underlying therapy-induced senescence in tumor and cardiovascular tissues. Anticancer therapies activate a conserved network of senescence-associated pathways in both tumor and cardiovascular tissues. Core mechanisms include persistent DNA damage response (DDR) activation, oxidative stress and mitochondrial dysfunction, p16^INK4a^/p21^CIP1^-mediated cell-cycle arrest, SASP-driven inflammatory signaling, and immune-metabolic remodeling involving cGAS/STING, mTOR/AMPK, and SIRT1 pathways. These interconnected processes promote the establishment and maintenance of therapy-induced senescence and facilitate SASP-mediated paracrine propagation. The resulting senescence-associated remodeling affects multiple tumor and cardiovascular target cell populations, contributing to chronic inflammation, tissue dysfunction, impaired repair responses, and long-term cardiovascular vulnerability. FigureLabs Plus (v4.1), an AI-powered scientific illustration platform, was used to assist with the generation of the graphical elements of this figure. The scientific content and graphical design were verified, reviewed, and finalized by the authors. Created with FigureLabs (https://figurelabs.ai).

The following sections discuss the principal molecular pathways underlying TIS and their implications in both cancer progression and cardiovascular remodeling.

### 2.1 DNA damage response and cell-cycle arrest pathways

A central hallmark of TIS is the persistent activation of the DDR following exposure to genotoxic stress induced by chemotherapy, radiotherapy, and targeted therapies. DNA lesions generated by these treatments activate ATM/ATR signaling cascade and downstream checkpoint mediators, including phosphorylation of histone H2AX and activation of checkpoint proteins CHK1 and CHK2. These events ultimately lead to stabilization of the tumor suppressor p53 and transcriptional activation of cyclin-dependent kinase inhibitor p21^CIP1/WAF1^. In parallel, senescence is reinforced through activation of the p16^INK4a/retinoblastoma (Rb) pathway, which suppresses CDK4/6 activity and inhibits E2F-dependent transcription required for cell-cycle progression. Together, these pathways establish a stable proliferative arrest that limits the propagation of damaged cells and represents one of the principal antitumor effects of TIS (7, 29).

Although these mechanisms were initially characterized in tumor cells, similar pathways are activated in cardiovascular tissues exposed to anticancer therapies. Anthracyclines, radiotherapy, and several targeted agents induce chronic DNA damage in ECs, cardiomyocytes, fibroblasts, and vascular smooth muscle cells, thereby promoting acquisition of a senescence-associated phenotype (1). In cardiovascular tissues, sustained activation of p53/p21 and p16/Rb signaling contributes to endothelial dysfunction, impaired regenerative capacity, vascular stiffness, myocardial remodeling, and fibrosis. Of note, even post-mitotic cardiomyocytes may undergo a senescence-like state characterized by persistent DNA damage signaling, mitochondrial dysfunction, altered metabolic homeostasis, impaired stress adaptation, and chronic inflammatory signaling (1). Notably, senescence is increasingly recognized as a dynamic rather than strictly irreversible process. Indeed, therapy-induced senescent tumor cells may persist after treatment and retain a degree of biological plasticity, potentially contributing to tumor relapses, stem-like reprogramming [e.g., senescence-associated stemness (SAS)], and therapeutic resistance. Persistent DDR signaling may therefore contribute not only to stable tumor growth arrest, but also to prolonged senescence-associated dysfunction within both tumor and cardiovascular tissues (29, 30).

### 2.2 NF-*κ*B signaling, SASP, and mitochondrial dysfunction

Beyond durable cell-cycle arrest, senescent cells acquire a complex senescence-associated secretory phenotype, characterized by the release of pro-inflammatory cytokines, chemokines, growth factors, extracellular vesicles, and matrix-remodeling enzymes. Persistent DDR signaling, together with cytoplasmic DNA sensing pathways such as cGAS/STING, promotes NF-*κ*B-dependent transcriptional reprogramming and chronic sterile inflammatory activation, which collectively represent major drivers of SASP production and senescence maintenance (31). Senescence-associated chromatin instability and formation of cytoplasmic chromatin fragments (CCFs) may further amplify cGAS/STING-dependent inflammatory signaling and NF-*κ*B activation, thereby reinforcing chronic SASP activity and propagation of the senescent phenotype (1). Key SASP mediators include IL-6, IL-8, TNF-α, MCP-1, insulin-like growth factor-binding protein 7 (IGFBP7), and matrix metalloproteinases, all of which profoundly contribute to persistent inflammatory signaling and microenvironmental reprogramming within the surrounding TME (7, 16, 29).

The biological consequences of SASP signaling are highly context dependent. In tumor tissues, transient SASP activity may reinforce senescence and facilitate immune-mediated clearance of damaged tumor cells. However, when senescent cells persist, chronic SASP signaling may promote sustained inflammation, extracellular matrix remodeling, immunosuppression, paracrine-propagated senescence, and TME dysfunction. In this setting, SASP-mediated inflammatory and paracrine signaling may progressively shift senescence from an initial tumor-suppressive response toward a tumor-promoting state associated with tumor plasticity, therapy resistance, and disease progression (16, 30, 32).

Importantly, the same inflammatory pathways are also activated in cardiovascular tissues following exposure to anticancer treatment. Senescent ECs, fibroblasts, and vascular smooth muscle cells release SASP mediators that promote endothelial dysfunction, vascular aging, myocardial fibrosis, and adverse cardiac remodeling (1). Chronic NF-*κ*B activation may further sustain persistent low-grade inflammatory activation associated with accelerated biological aging and cardiovascular dysfunction (31). Similarly, cGAS/STING-dependent inflammatory signaling may also contribute to chronic cardiovascular remodeling in tissues exposed to anticancer therapies (1, 31).

Mitochondrial dysfunction and oxidative stress further contribute to the establishment and maintenance of TIS in both tumor and cardiovascular tissues. Senescent cells exhibit altered mitochondrial morphology, impaired oxidative phosphorylation, increased lysosomal activity, and excessive production of reactive oxygen species (ROS), which amplify DNA damage and inflammatory signaling through self-sustaining feedback loops (1, 7).

These mechanisms are particularly relevant in cardiovascular tissues, where mitochondrial homeostasis is essential for energetic balance and contractile function. In cardiomyocytes, mitochondrial dysfunction impairs ATP production and calcium handling while simultaneously promoting pro-fibrotic and pro-inflammatory pathways. Similarly, oxidative stress in ECs reduces nitric oxide (NO) bioavailability and accelerates vascular aging (27). Thus, mitochondrial dysfunction, oxidative stress and chronic inflammatory signaling represent a shared mechanistic denominator linking TIS in tumor cells with therapy-associated cardiovascular remodeling.

### 2.3 mTOR/AMPK signaling and metabolic rewiring

Senescent cells undergo profound metabolic adaptations that support long-term survival, chronic inflammatory signaling, and maintenance of the senescent phenotype (33). Among the principal regulators of these metabolic alterations are the mechanistic target of rapamycin (mTOR) and AMP-activated protein kinase (AMPK) pathways, which coordinately regulate cellular energy balance, nutrient sensing, autophagy, and cellular stress responses (7, 32).

mTOR signaling promotes anabolic metabolism, increased protein synthesis, cellular enlargement, and translation of inflammatory SASP components, thereby reinforcing senescence maintenance and persistent inflammatory activity (7, 32). Sustained mTOR activation has been associated with impaired autophagic flux and accumulation of dysfunctional mitochondria, further amplifying oxidative stress and inflammatory signaling. In this context, mTOR acts not only as a metabolic regulator, but also as an important amplifier of the senescence-associated secretory and inflammatory phenotype.

Conversely, AMPK acts as a cellular energy sensor activated under conditions of metabolic stress and increased AMP/ATP ratios. Through regulation of mitochondrial quality control, oxidative metabolism, autophagy, and p53-related stress responses, AMPK contributes to cellular adaptation to energetic stress while limiting excessive anabolic signaling. The balance between mTOR and AMPK signaling therefore critically regulates the intensity, stability and biological consequences of TIS across different cellular contexts (1, 7). In parallel, altered activity of longevity-associated regulators such as SIRT1, may further contribute to mitochondrial dysfunction, impaired stress adaptation, and persistence of the senescent phenotype across both tumor and cardiovascular tissues (1, 34).

Notably, dysregulation of these pathways has been observed in both senescent tumor cells and cardiovascular tissues exposed to anticancer therapies. In cardiovascular cells, chronic mTOR activation contributes to hypertrophy, fibrosis, metabolic dysfunction, vascular remodeling, and impaired autophagy, whereas impaired AMPK signaling compromises mitochondrial quality control, energetic homeostasis and stress adaptation. These observations suggest that metabolic rewiring contributes to the persistence and functional consequences of TIS across both tumor and cardiovascular tissues. However, because mTOR, AMPK, and related metabolic pathways also participate in tumor suppression, tissue repair, immune regulation, and physiological adaptation to stress, their therapeutic modulation requires careful contextualization. Excessive or poorly timed inhibition of senescence-associated metabolic signaling could theoretically interfere with antitumor efficacy or adaptive tissue responses. Therefore, strategies aimed at modulating mTOR/AMPK/SIRT1-related pathways should seek to attenuate persistent pathological senescence while preserving transient senescence programs required for tumor control and tissue homeostasis.

Collectively, these interconnected pathways highlight the multifaceted biological nature of TIS and its potential contribution to both tumor-associated and cardiovascular remodeling. Understanding how metabolic and nutrient-sensing mechanisms operate across BC and cardiovascular tissues may therefore provide important insights into the pathogenesis of therapy-associated cardiovascular complications and their long-term clinical consequences. These observations have stimulated increasing interest in senescence-targeting therapeutic strategies aimed at modulating persistent senescence-associated remodeling, as discussed in subsequent sections.

## 3 Therapy-induced senescence and tumor remodeling in breast cancer

TIS represents a major cellular response to anticancer treatments in BC, particularly following exposure to chemotherapy, radiotherapy, and targeted agents (35–39). Historically, TIS was regarded as a stable and essentially irreversible tumor growth arrest and therefore as a desirable therapeutic outcome alternative to apoptosis or tumor-cell death (40). This view has been challenged by more recent evidence demonstrating that senescent cancer cells remain metabolically active, can remodel the surrounding TME, and, under specific conditions, may escape senescence-associated arrest and acquire stem-like or more aggressive phenotypes (29, 41). Accordingly, several therapeutic modalities can promote a senescence-like state characterized by proliferative arrest, increased senescence-associated *β*-galactosidase (SA-*β*-Gal) activity, persistent DDR activation and acquisition of a metabolically active phenotype. In this setting, TIS should be regarded not simply as a terminal response to treatment, but as a biologically active and context-dependent state that may shape both immediate therapeutic response and long-term tumor evolution.

Among conventional anticancer therapies, chemotherapy represents one of the best characterized inducers of TIS. Anthracyclines, particularly doxorubicin, strongly activate DDR signaling through induction of DNA double-strand breaks, topoisomerase II inhibition, oxidative stress, mitochondrial injury, thereby promoting activation of p53/p21 and p16^INK4a^/Rb pathways and subsequent senescence-associated growth arrest (16, 42). Other cytotoxic agents used in BC treatment, including cisplatin, paclitaxel, and temozolomide can also induce senescence through persistent mitotic stress, genotoxic damage and downstream activation of senescence-associated pathways (16). Importantly, the cellular outcome of chemotherapy exposure depends on drug dose, duration, treatment schedule and cellular context: high levels of damage may lead to apoptosis, whereas lower or intermediate levels of persistent stress may preferentially induce a senescence-associated phenotype (16, 39–41). In this sense, TIS may represent a relevant component of the therapeutic response in BC, particularly in tumors exposed to prolonged genotoxic, mitotic or oxidative stress.

Radiotherapy is another major inducer of senescence in BC cells. Ionizing radiation promotes DNA double-strand breaks and persistent DDR activation through ATM/ATR signaling pathways, which may culminate in durable cell-cycle arrest when DNA damage is not fully repaired (36, 43). Although this response contributes to local tumor growth control, irradiated senescent cells may remain metabolically active and influence the TME through chronic secretory, inflammatory and remodeling programs. Thus, radiation-induced senescence should be considered not only as a mechanism of local tumor suppression, but also as a biologically active state with potential long-term consequences for tumor behavior, stromal remodeling, immune regulation, and tissue homeostasis. In this context, recent evidence demonstrated that radiotherapy in BC is associated with activation of senescence-related transcriptional and inflammatory programs, further supporting the role of TIS as a major component of the biological response to irradiation (37).

Targeted therapies may also induce or reinforce senescence-like programs in BC. This is particularly relevant for CDK4/6 inhibitors, such as palbociclib, ribociclib, and abemaciclib, which are widely used in hormone receptor–positive BC and act by blocking cell-cycle progression at the G1/S transition. By directly targeting the cyclin D–CDK4/6–Rb axis, these agents can induce durable cell-cycle arrest and senescence-like features in tumor cells, thereby contributing to sustained growth suppression (36). However, the biological consequences of CDK4/6 inhibitor-induced senescence are context dependent, as persistent senescent cells may also acquire secretory and adaptive programs with potential implications for tumor persistence and therapeutic resistance. In HER2-positive BC, inhibition of the EGFR/HER2 signaling axis through agents such as lapatinib has also been associated with induction of senescence-like states, suggesting that TIS may contribute, at least in part, to the therapeutic activity of HER2-targeted strategies (38, 44).

Overall, these observations indicate that TIS is not a marginal or incidental response to BC therapy, but rather a central biological outcome of multiple treatment modalities. By integrating cell-cycle arrest, persistent stress signaling, secretory reprogramming, and microenvironmental remodeling, TIS may profoundly influence both tumor control and subsequent tumor evolution. This dual nature provides the conceptual basis for interpreting senescence as both an initially beneficial therapeutic response and a potential driver of long-term tumor plasticity, resistance, and recurrence. Importantly, the susceptibility to TIS and its biological consequences are highly cell type-, tissue-, and context-dependent, reflecting differences in proliferative capacity, metabolic state, DNA repair competence, and microenvironmental interactions across both tumor and cardiovascular cell populations (7, 8).

### 3.1 Beneficial effects of TIS: tumor suppression and immune-mediated clearance

In BC, TIS may initially exert beneficial antitumor effects by establishing a stable cell-cycle arrest that limits the proliferative capacity of damaged tumor cells, thereby restricting clonal expansion and tumor progression (16, 45). In this early tumor-suppressive context, senescence can represent an alternative therapeutic outcome to apoptosis, particularly in response to chemotherapy, radiotherapy, and CDK4/6 inhibition.

Importantly, the antitumor effects of TIS are not exclusively cell-autonomous. Senescent tumor cells actively communicate with the surrounding microenvironment through the SASP, which can modulate immune surveillance and tissue remodeling (7). Although persistent SASP signaling may exert detrimental long-term effects, transient SASP activity during the early phases of senescence may facilitate immune-mediated recognition and clearance of damaged tumor cells. This temporal distinction is central to the dual role of TIS: an acute and properly resolved senescence response may support tumor suppression, whereas chronic persistence of senescent cells may progressively acquire pathological consequences.

Several SASP components contribute to the recruitment and activation of immune populations involved in senescence surveillance, including macrophages, natural killer (NK) cells, neutrophils, and T lymphocytes (16, 17). In addition, senescence-associated inflammatory signaling may enhance IFN*γ*-mediated responses and antigen presentation pathways, thereby facilitating immune recognition of damaged tumor cells and promoting tumor regression under specific conditions. These observations support the concept that transient senescence-associated signaling may contribute to physiological antitumor responses, immune-mediated tumor clearance and tissue homeostasis. Clinical evidence further supports the antitumor relevance of TIS, as senescence-associated phenotypes have been identified in approximately 40% of BC samples following neoadjuvant chemotherapy (39, 40, 46, 47).

The beneficial role of TIS appears particularly relevant in the context of targeted therapies such as CDK4/6 inhibitors, where induction of a senescence-like phenotype may contribute to prolonged tumor control and durable suppression of cell proliferation in hormone receptor–positive breast cancer (36). Similarly, radiotherapy-induced senescence may enhance local tumor control by preventing proliferation of cells harboring extensive DNA damage. In these settings, TIS may therefore function as an adaptive stress-response program that complements direct cytotoxicity and contributes to therapeutic efficacy.

Collectively, these findings support the view that TIS initially acts as an adaptive and tumor-suppressive response capable of limiting malignant progression and facilitating immune-mediated tumor clearance. However, the biological benefit of TIS depends on its timely resolution and effective immune surveillance. When senescent cells persist and chronic SASP production is sustained, this initially protective program may progressively shift toward a pathological state favoring tumor plasticity, microenvironment remodeling and disease recurrence.

### 3.2 Detrimental effects of persistent senescence in BC

Although TIS may initially contribute to tumor suppression, increasing evidence indicates that the long-term persistence of senescent tumor and stromal cells may paradoxically promote tumor progression, therapeutic resistance, and disease recurrence (36, 48, 49). In this context, senescence should not be regarded as a biologically inert endpoint, but rather as a dynamic and context-dependent state capable of profoundly influencing tumor behavior over time.

One of the major detrimental consequences of persistent TIS is the acquisition of tumor cell plasticity and stem-like features (22, 29). Senescent tumor cells may remain in a dormant or slow-cycling state for prolonged periods and, under specific conditions, escape senescence-associated growth arrest and re-enter the cell cycle, generating more aggressive and therapy-resistant cellular populations (30, 41). This phenomenon, often referred to as SAS, has been linked to tumor relapse, metastatic dissemination, and resistance to subsequent therapies (36).

In BC survivorship, this mechanism may be clinically relevant because persistent therapy-induced senescent tumor cells could contribute, together with other mechanisms of tumor dormancy and immune escape, to minimal residual disease and late relapse. This is particularly important in early-stage BC, where residual disease may persist after apparently effective treatment and late recurrences can occur years or even decades after diagnosis, with risk varying according to tumor stage, biological subtype, nodal status, and treatment exposure (3, 50). In this setting, the ability of a subset of TIS cells to overcome proliferative arrest may provide a biologically plausible link between treatment-induced stress responses, tumor dormancy, and delayed disease recurrence. Persistent SASP signaling and senescence-associated remodeling have also been implicated in therapeutic resistance and aggressive tumor behavior in specific BC subtypes, including HER2-positive and triple-negative BCs (51). These effects may be mediated not only by senescent tumor cells, but also by senescent stromal and immune components within the breast tumor microenvironment. Through chronic secretion of cytokines, chemokines, growth factors, matrix-remodeling enzymes, and extracellular vesicles, persistent senescent cells may create a tissue context that supports tumor cell survival, phenotypic adaptation, and resistance to subsequent therapies.

Importantly, many of these detrimental effects are sustained through chronic SASP-mediated remodeling of the TME (7, 52), which promotes inflammatory activation, extracellular matrix remodeling, angiogenesis, EMT, immune suppression and paracrine propagation of senescence to neighbouring cells, thereby creating conditions favorable to tumor progression (45, 53–55). Thus, while TIS may initially suppress tumor growth, persistence of senescent tumor and stromal cells may progressively reshape the breast TME toward a pro-tumorigenic state associated with tumor plasticity, metastatic progression, therapeutic resistance, and disease recurrence.

### 3.4 SASP-driven remodeling of the breast tumor microenvironment

Within the breast TME, persistent senescence-associated signaling profoundly reshapes intercellular communication, stromal organization, and immune regulation, thereby influencing tumor progression and metastatic evolution. Senescent tumor and stromal cells actively remodel surrounding tissues through secretion of cytokines, chemokines, growth factors, proteases, and extracellular matrix-remodeling enzymes (7). The biological consequences of this secretory activity are highly context-dependent and may vary according to cell type, tissue origin, senescence-inducing stimulus, treatment exposure, and timing of senescent cell persistence.

In BC, persistent SASP signaling may profoundly alter intercellular communication and contribute to the establishment of a permissive tumor-promoting microenvironment. SASP-associated mediators such as IL-6, IL-8, VEGF, TGF-*β*, and matrix metalloproteinases (MMPs) have been implicated in angiogenesis, extracellular matrix degradation, EMT, and increased tumor cell invasiveness (53, 55). In particular, pro-angiogenic SASP mediators such as VEGF may promote neovascularization and sustain tumor growth within the senescent TME (16, 56). These mediators may also reinforce paracrine communication between tumor cells and stromal compartments, thereby amplifying local inflammatory and remodeling programs.

Importantly, senescence-associated remodeling also affects stromal and immune compartments within the TME. Senescent fibroblasts may acquire cancer-associated fibroblast (CAF)-like properties and contribute to extracellular matrix remodeling, chronic inflammatory activation, and metastatic niche formation (8, 56–59). In parallel, SASP-associated remodeling may promote recruitment or functional polarization of immunosuppressive immune populations, thereby impairing effective antitumor immune surveillance and facilitating immune escape and tumor persistence (60). Recent evidence suggests that TIS cancer cells (TISCCs) actively regulate tumor ecosystem dynamics through bidirectional interactions with immune and stromal populations, progressively shifting the TME from immune-mediated tumor control toward chronic immunosuppression and therapeutic resistance (61). In this context, the senescent and “aged” TME should no longer be considered as a passive bystander, but rather as an active participant in BC progression and metastatic evolution.

Recent studies further suggest that SASP composition itself may influence tumor behavior in highly dynamic ways. Specific SASP components released by senescent cells, including GM-CSF and bFGF, have been shown to enhance tumor cell migration and dissemination through activation of pro-invasive signaling pathways such as JNK signaling (62). These findings reinforce the idea that senescent cells may actively shape tumor evolution not only through direct effects on malignant cells, but also through broad reprogramming of the surrounding stromal and immune microenvironment. Importantly, many of the senescence-associated pathways and inflammatory mediators involved in BC remodeling are not restricted to malignant tissues but may also affect distant non-malignant organs exposed to anticancer therapies. These observations further support the concept that persistent senescence-associated signaling may extend beyond the local TME and contribute to systemic tissue dysfunction and therapy-related cardiovascular remodeling.

## 4 Therapy-induced senescence in cardiovascular tissues

Beyond their antitumor activity, several anticancer therapies have been shown to induce premature senescence in multiple cardiovascular cell populations, thereby promoting persistent structural and functional alterations within the cardiovascular system. Anthracyclines, radiotherapy, targeted therapies, and emerging immune-activating therapies can induce senescence-like programs in cardiovascular tissues through persistent cellular stress and sustained damage signaling (5, 23, 63). Importantly, these stressors activate molecular programs similar to those observed in tumor cells undergoing TIS, ultimately promoting persistent senescence-associated dysfunction within cardiovascular tissues (5, 8).

Among cardiovascular cell populations, ECs appear particularly vulnerable to TIS. Exposure to anthracyclines and ionizing radiation promotes persistent oxidative stress, impaired NO bioavailability, reduced angiogenic potential, and chronic endothelial dysfunction, leading to acquisition of a pro-inflammatory endothelial phenotype associated with impaired vascular homeostasis (23). Endothelial senescence and the resulting endothelial dysfunction is increasingly recognized as one of the earliest manifestations of anthracycline-induced cardiovascular injury and a key initiating event in the establishment and propagation of senescence-associated cardiovascular remodeling (5, 23, 42, 64, 65). Consistently, experimental evidence supports the role of oxidative stress-driven endothelial senescence in vascular dysfunction and premature vascular remodeling, highlighting senescence-associated pathways as critical mediators of cardiovascular injury (27). Endothelial senescence is increasingly recognized as a major contributor to therapy-associated cardiovascular dysfunction and accelerated vascular aging in cancer survivors (23, 55). Within this framework, the relevance of radiation-induced senescence lies not in a unique susceptibility of ECs to DNA damage, but in the potential for persistent impairment of NO signaling, angiogenic repair and vascular homeostasis following ionizing radiation exposure (1).

Consistent with their established role in TIS within tumor cells, anthracyclines, particularly doxorubicin, represent one of the most extensively studied inducers of cardiovascular senescence. Doxorubicin has been shown to induce senescence in ECs, cardiomyocytes, vascular smooth muscle cells (VSMCs), cardiac progenitor cells, and fibroblasts through excessive ROS generation, mitochondrial impairment, telomere dysfunction, and sustained DDR activation (5). Importantly, experimental studies demonstrated that low and moderate doses of doxorubicin preferentially induce a senescence-associated phenotype rather than apoptotic cell death in cardiovascular cells, leading to persistent cellular dysfunction and chronic tissue remodeling (5, 66, 67). Likewise, radiation exposure promotes endothelial and myocardial senescence through oxidative DNA damage, mitochondrial dysfunction, and chronic inflammatory activation, thereby contributing to impaired vascular repair, progressive myocardial remodeling, and delayed cardiovascular toxicity (68, 69). Similar senescence-associated endothelial alterations have also been observed following exposure to antimetabolites such as 5-fluorouracil, which may induce endothelial dysfunction through oxidative stress, impaired NO signaling, and suppression of SIRT1-associated protective pathways (70).

In addition to ECs, TIS also affects cardiomyocytes, fibroblasts, and VSMCs. Senescent cardiomyocytes exhibit mitochondrial dysfunction, altered calcium handling and impaired contractile properties. Unlike proliferative cell populations, cardiomyocytes may undergo a post-mitotic senescence-like state characterized by mitochondrial dysfunction, telomere-associated DNA damage, and maladaptive secretory signaling despite their limited proliferative capacity (1). In parallel, senescent fibroblasts and VSMCs contribute to extracellular matrix remodeling, myocardial fibrosis, vascular calcification, arterial stiffness, and impaired vascular compliance (5, 71).

Emerging evidence further suggests that senescence-associated cardiovascular remodeling may persist long after treatment completion, supporting the concept that cancer therapies can induce a chronic premature aging phenotype within cardiovascular tissues rather than solely a transient toxic insult. In this context, the dual and context-dependent nature of senescence within cardiovascular tissues may resemble a “Yin and Yang” process, exerting adaptive effects in the acute phase while contributing to chronic dysfunction and adverse remodeling over time (72). Although cardiovascular tissues are not the intended therapeutic target, their vulnerability partly reflects the activation of stress and senescence pathways shared with tumor cells. Cardiovascular TIS should therefore be regarded as an unintended consequence of anticancer treatment that may contribute to the transition from acute injury to chronic endothelial, myocardial, and vascular remodeling (Table 1).

**Table 1:** Anticancer therapies associated with therapy-induced cardiovascular senescence and remodeling.

Therapy class	Cardiovascular target cells/tissues	Main senescence-associated mechanisms	Major cardiovascular consequences	Representative references
Anthracyclines *(e.g., doxorubicin)*	Endothelial cells, cardiomyocytes, VSMCs, cardiac progenitor cells, fibroblasts	ROS accumulation; persistent DDR activation; mitochondrial dysfunction; telomere dysfunction; p53/p21 and p16/Rb activation; SASP amplification	Endothelial dysfunction; myocardial fibrosis; impaired regenerative capacity; vascular aging; LV dysfunction and HF phenotypes	(2, 5, 63, 66)
Radiotherapy	Endothelial cells, cardiomyocytes, stromal cells, vascular wall	DNA double-strand breaks; oxidative stress; mitochondrial injury; chronic inflammatory activation; SASP propagation; radiation-induced bystander effect	Endothelial dysfunction; impaired vascular repair; vascular stiffness; myocardial fibrosis; delayed cardiovascular toxicity	(1, 2, 5, 63, 69)
HER2-targeted therapies	Cardiomyocytes, endothelial cells, myocardial tissue	Impaired HER2-mediated survival signaling; Mitochondrial dysfunction; Oxidative stress; Impaired stress adaptation; Senescence-associated myocardial remodeling	LV dysfunction; impaired myocardial repair; Progressive myocardial remodeling; Increased cardiovascular vulnerability	(1, 5, 179)
CDK4/6 inhibitors	Endothelial cells, vascular tissues, tumor and stromal compartments	Reinforcement of cell-cycle arrest; p16/p21 pathway modulation; senescence-like phenotype induction; Immune and SASP-associated remodeling	Potential endothelial dysfunction; altered vascular repair; possible contribution to tissue aging	(36, 63, 180, 181)
Tyrosine kinase inhibitors *(e.g., ponatinib, axitinib)*	Endothelial cells, vascular wall	Oxidative stress; endothelial injury; inflammatory activation; senescence-associated endothelial dysfunction	Hypertension; vascular dysfunction; accelerated atherosclerosis; pro-thrombotic vascular phenotype	(1, 2, 26, 63, 179)
Endocrine therapies	Vascular endothelium, cardiometabolic tissues	Hormonal deprivation; oxidative stress; metabolic dysfunction; endothelial impairment; possible senescence-associated vascular remodeling	Vascular dysfunction; increased cardiometabolic risk; accelerated vascular aging; increased cardiovascular vulnerability	(1, 4, 182)
Immune checkpoint inhibitors/immune-activating therapies	Endothelial cells, immune cells, myocardium, vascular tissues	Chronic immune activation; SASP amplification; immune–vascular crosstalk; inflammaging-like remodeling	Vascular inflammation; myocarditis-related injury; endothelial dysfunction; accelerated cardiovascular aging	(26, 49, 63, 146)
Antimetabolites *(5-FU/capecitabine)*	Endothelial cells, vascular endothelium	p38/JNK activation; suppression of eNOS and SIRT1; oxidative stress; impaired NO signaling	Endothelial dysfunction; vasospasm; ischemic injury; vascular aging	(63, 70)
Platinum-based agents *(e.g., cisplatin)*	Endothelial cells, vascular tissues	ROS accumulation; mitochondrial dysfunction; DNA damage; endothelial injury; senescence-associated inflammatory signaling	Endothelial injury; vascular inflammation; impaired vascular repair; accelerated vascular aging	(2, 63, 183)
Taxanes *(e.g., docetaxel, paclitaxel)*	Endothelial cells Vascular tissues Stromal and mesenchymal cells	Cell-cycle arrest Microtubule stabilization Oxidative stress Mitochondrial dysfunction Senescence-like phenotype induction	Endothelial dysfunction; Increased arterial stiffness; Arrhythmias; vascular remodeling	(4, 63, 184)

### 4.1 Senescence-associated cardiovascular remodeling and dysfunction

The accumulation of senescent cardiovascular cells following anticancer treatment promotes a broad spectrum of structural and functional alterations that progressively impair cardiovascular homeostasis. Rather than representing a transient toxic response, TIS contributes to a persistent remodeling program characterized by endothelial dysfunction, myocardial fibrosis, vascular stiffening, and progressive cardiovascular dysfunction. Over time, these alterations may favor the development of clinically relevant cardiovascular phenotypes, including heart failure, accelerated vascular aging, atherosclerotic disease, and chronic cardiovascular vulnerability in cancer survivors (1, 5, 26).

One of the earliest manifestations of cardiovascular TIS is endothelial dysfunction. Reduced NO bioavailability, chronic oxidative stress, impaired angiogenic capacity, and sustained inflammatory activation collectively disrupt vascular homeostasis and promote vascular dysfunction (23). Chronic endothelial activation further enhances leukocyte adhesion, vascular inflammation, and pro-atherogenic signaling, thereby contributing to accelerated vascular aging and atherosclerotic progression in cancer survivors (24). More broadly, experimental elimination of senescent intimal foam cells reduced atherosclerotic plaque burden and pathological plaque remodeling, providing direct evidence that senescent vascular-cell populations contribute causally to atherosclerotic progression (73). In this context, oxidative stress-driven endothelial senescence has been strongly associated with premature vascular remodeling and endothelial impairment, highlighting the central contribution of senescence-associated pathways to cardiovascular injury (27). Persistent endothelial dysfunction may further impair vascular repair mechanisms and promote pro-thrombotic vascular remodeling long after treatment completion (24). Supporting a mechanistic relationship between coagulation signaling and endothelial senescence, thrombin directly induced a senescence-associated phenotype in atrial endothelial cells and promoted angiotensin II-dependent pro-remodeling responses in a non-oncological experimental model (24, 74). However, whether this pathway contributes to persistent vascular remodeling following anticancer treatment remains to be established.

Beyond vascular dysfunction, persistent senescence-associated remodeling profoundly affects myocardial structure and function. Mitochondrial dysfunction and sustained cellular stress contribute to impaired myocardial adaptability, maladaptive remodeling, and progressive decline in cardiac performance. In parallel, profibrotic pathways activated within senescent cardiovascular tissues promote extracellular matrix deposition and myocardial fibrosis, thereby favoring ventricular stiffness and heart failure phenotypes (5, 71). Persistence of senescent fibroblasts and myofibroblasts, together with sustained TGF-*β*-mediated profibrotic signaling and extracellular matrix remodeling, may further impair reparative responses and contribute to progressive myocardial stiffening and adverse ventricular remodeling (1). Experimental evidence further suggests that chronic senescence-associated myocardial remodeling may impair reparative and regenerative responses following tissue injury, thereby favoring maladaptive repair programs, and amplifying long-term cardiac vulnerability after anticancer treatment (63). Persistent endothelial and microvascular dysfunction may additionally impair myocardial perfusion and tissue oxygen delivery, thereby contributing to progressive myocardial stiffening, impaired relaxation, and impaired cardiac reserve. These alterations may be particularly relevant in the development of heart failure phenotypes characterized by impaired ventricular compliance, endothelial dysfunction, microvascular impairment, low-grade inflammation, and accelerated biological aging. In this context, the senescence-associated cardiovascular phenotype shares several pathophysiological features with heart failure with preserved ejection fraction (HFpEF), including vascular stiffness, impaired myocardial relaxation, chronic inflammatory activation, and microvascular endothelial dysfunction. These observations suggest that TIS may contribute to the premature emergence of HFpEF-like syndromes in cancer survivors, particularly in patients exposed to multimodal anticancer therapies and in those with pre-existing cardiometabolic vulnerability (1, 5, 26, 75). This concept is further supported by emerging clinical evidence indicating that HFpEF phenotypes may be more prevalent than heart failure with reduced ejection fraction (HFrEF) among breast cancer survivors, particularly following contemporary multimodal anticancer therapies, possibly reflecting the contribution of chronic endothelial and microvascular senescence-associated dysfunction to progressive cardiovascular remodeling (4, 63, 76, 77).

TIS also contributes to vascular stiffness and accelerated vascular aging. Senescence-associated alterations within VSMCs promote extracellular matrix remodeling, osteogenic reprogramming, vascular calcification, and loss of vascular elasticity, ultimately leading to arterial stiffening and impaired vascular compliance (71). These changes may further increase afterload, impair vascular compliance and intercellular communication within the vascular wall, by contributing to the progressive cardiovascular vulnerability observed in cancer survivors.

Collectively, these senescence-associated alterations may ultimately translate into clinically relevant cardiovascular phenotypes increasingly recognized in cancer survivors, including progressive left ventricular dysfunction, heart failure, accelerated atherosclerosis, vascular stiffness, hypertension, microvascular dysfunction, and thrombotic complications (1, 4, 5, 24, 26, 63). Altogether, these findings support the concept that cardiovascular TIS is not merely an epiphenomenon of anticancer treatment, but rather a biologically active process contributing to progressive cardiovascular dysfunction, adverse tissue remodeling and long-term survivorship vulnerability.

### 4.2 SASP, inflammaging and long-term cardiovascular risk

Beyond its cell-autonomous effects, TIS exerts profound systemic consequences through chronic SASP production and sustained inflammatory remodeling. Senescent cardiovascular and non-cardiovascular cells release a broad spectrum of pro-inflammatory cytokines, chemokines, growth factors, matrix-remodeling enzymes, and pro-fibrotic mediators capable of sustaining chronic low-grade inflammation and progressive cardiovascular remodeling (7, 26). Direct evidence supports this concept: circulating pro-inflammatory cytokines increase by 15%–48% following adjuvant chemotherapy in postmenopausal BC patients, although most return close to pre-treatment levels within 6 months of completing treatment (78). However, this inflammatory response is not uniform. In a cohort of 734 BC survivors, mean C-reactive protein (CRP) levels remained above the clinical inflammation cutoff at 31 months post-diagnosis, correlating with poorer overall and disease-free survival (79). This heterogeneity suggests that while treatment-induced inflammation is transient in most patients, a subset may experience more sustained low-grade activation capable of contributing to early vascular remodeling (79). In cancer survivors, this senescence-associated inflammatory environment may therefore contribute to accelerated vascular aging and delayed cardiovascular complications after treatment completion. Multiple anticancer therapies commonly used in BC — including anthracyclines, radiotherapy, HER2-targeted therapies, CDK4/6 inhibitors, tyrosine kinase inhibitors (TKIs) and endocrine therapies — have all been associated, to varying degrees, with cardiovascular remodeling, vascular dysfunction, and senescence-associated stress responses (1). Anthracyclines and radiotherapy remain the best characterized inducers of cardiovascular senescence, primarily through DNA damage response (DDR) activation, oxidative stress, and mitochondrial dysfunction. By contrast, targeted and endocrine therapies appear to promote senescence-associated cardiovascular remodeling through more heterogeneous, therapy-specific, and context-dependent molecular mechanisms involving altered survival signaling, endothelial dysfunction, metabolic dysregulation, and impaired stress adaptation. Accordingly, TKIs such as ponatinib and axitinib have been associated with endothelial senescence, oxidative stress, and vascular dysfunction, whereas trastuzumab-related cardiotoxicity may involve impaired cardiomyocyte stress adaptation and mitochondrial dysfunction (1). Despite these therapy-specific differences, many of these upstream pathways ultimately converge on shared senescence-associated mechanisms, including persistent DDR activation, mitochondrial dysfunction, chronic SASP signaling, and inflammatory remodeling, as discussed in Sect. 2. Accordingly, the cardiovascular effects depicted in Figure 1 should not be interpreted as uniformly direct consequences of TIS. Cardiomyocyte death, microvascular injury, and treatment-related metabolic or endocrine alterations may occur as direct consequences of anticancer therapy without requiring the establishment of a senescence program. TIS may also directly contribute to specific cellular alterations, including endothelial dysfunction, impaired regenerative capacity, and persistent pro-inflammatory and pro-fibrotic SASP signaling. More complex cardiovascular outcomes, including myocardial remodeling, fibrosis, vascular stiffness, atherosclerosis, and thrombotic risk, are multifactorial phenomena and may arise from direct therapy-related injury while being further exacerbated by persistent TIS and SASP-mediated inflammation. These categories are not mutually exclusive, because the same therapeutic insult may simultaneously induce an acute cellular injury and promote the establishment of a senescent phenotype.

In the latter context, persistent low-grade inflammatory signaling may amplify endothelial dysfunction, reduce NO bioavailability, promote pro-coagulant and pro-thrombotic vascular phenotypes, and reinforce myocardial and vascular remodeling long after anticancer treatment has ended (4, 26). These mechanisms provide a plausible biological explanation for the delayed emergence of cardiovascular complications observed in BC survivorship, particularly in patients exposed to cumulative multimodal therapies or with pre-existing cardiovascular risk factors. The clinical relevance of this vulnerability is substantial. In a population-based cohort of women aged 66–80 years with early-stage BC, each additional decade of age was associated with a 79% increase in the overall risk of congestive heart failure (HR, 1.79; 95% CI, 1.66–1.93). Within the same cohort, anthracycline-treated women aged 66–70 years had a 26% higher risk than those receiving non-anthracycline chemotherapy (HR, 1.26; 95% CI, 1.12–1.42) (80). Anthracycline cardiotoxicity is also strongly dose-dependent, increasing from approximately 3%–5% at cumulative doxorubicin doses ≤400 mg/m^2^ to nearly 50% at doses around 700 mg/m^2^ (81, 82), while trastuzumab further increases the risk of cancer therapy-related cardiac dysfunction, particularly following prior anthracycline exposure (83).

Importantly, the long-term cardiovascular burden observed in cancer survivors is multifactorial and cannot be attributed to TIS alone. Rather than acting independently, the direct therapy-related and senescence-associated mechanisms outlined above may interact with neurohormonal alterations and pre-existing cardiovascular risk factors to accelerate the biological aging of the cardiovascular system, a process increasingly conceptualized as cancer therapy-related cardiovascular aging (CTR-CVA) (1, 4, 5, 63). This framework is particularly relevant to younger BC patients with long life expectancy after treatment. Long-term observations in childhood cancer survivors, including those exposed to chest radiotherapy, demonstrate substantially increased risk of myocardial infarction and congestive heart failure compared with their siblings, confirming that therapy-related cardiovascular vulnerability may persist for decades (63, 84).

However, the observed association between prior cancer therapy and later myocardial infarction or heart failure does not, by itself, establish TIS as the underlying mechanism, since alternative and non-mutually exclusive pathways may also contribute to long-term cardiovascular injury. Anthracyclines can directly damage cardiomyocytes through topoisomerase-II*β*-mediated DNA double-strand breaks and mitochondrial dysfunction independently of a senescence program (85), while cumulative cardiomyocyte loss and radiation-induced microvascular injury may further promote progressive myocardial and vascular damage. Within this broader framework, TIS is implicated as a potential, but not exclusive, contributor because anticancer treatments induce persistent senescence-associated phenotypes in cardiovascular cells, while pharmacological clearance of senescent cells attenuated doxorubicin-induced cardiotoxicity and improved cardiac function in mice (66, 67, 70, 86).

Nevertheless, direct evidence linking cardiovascular TIS to late clinical outcomes in humans remains lacking. TIS should therefore be regarded as a candidate mechanistic bridge — rather than the sole explanation — linking cancer treatment, chronic inflammaging, systemic tissue remodeling, and long-term cardiovascular vulnerability. This cardiovascular-focused perspective provides the basis for the broader systemic crosstalk discussed in the following section.

## 5 Therapy-induced senescence and systemic (biological) crosstalk

The remarkable overlap between senescence-associated pathways activated in tumor and cardiovascular tissues suggests that TIS should be regarded as a systemic and interconnected biological process rather than an isolated treatment-related event. Although senescence has historically been investigated within distinct pathological contexts, many of the molecular and cellular pathways activated in response to anticancer therapies appear to be remarkably conserved across tumor and cardiovascular tissues. These include persistent DNA damage signaling, mitochondrial dysfunction, oxidative stress, SASP production, inflammatory activation, immune remodeling, and metabolic adaptation.

Beyond their direct intracellular effects, senescence-associated pathways profoundly alter intercellular communication and reshape tissue microenvironments through chronic inflammatory, metabolic, immune-mediated, and vascular crosstalk (5, 7, 8, 26). As a consequence, TIS may simultaneously influence tumor persistence, cardiovascular remodeling, immune dysregulation, and accelerated tissue aging, thereby extending its biological impact far beyond the initially damaged tissue compartment. This systemic perspective provides the rationale for considering TIS as a biological continuum connecting tumor remodeling, cardiovascular dysfunction, inflammaging, and long-term survivorship vulnerability.

### 5.1 SASP, inflammaging and immune-mediated senescence propagation

Beyond local tissue remodeling, persistent senescence-associated signaling profoundly influences systemic immune homeostasis and chronic inflammatory responses. The interaction between senescent cells, immune surveillance, and inflammaging has emerged as a central mechanism through which TIS may propagate tissue dysfunction beyond the initially damaged compartment.

Persistent SASP-mediated paracrine signaling may amplify tissue dysfunction by propagating senescence-associated phenotypes across multiple cellular compartments, thereby establishing a self-perpetuating cycle of chronic remodeling and inflammatory activation. Increasing evidence suggests that chronic senescence-associated inflammatory signaling promotes widespread intercellular and inter-organ communication capable of propagating tissue dysfunction far beyond the initially damaged microenvironment (7, 87). Similar mechanisms may also occur following radiation exposure through the radiation-induced bystander effect (RIBE), in which irradiated cells propagate oxidative stress, inflammatory signaling, and senescence-associated remodeling to adjacent non-irradiated tissues via paracrine mediators, cytokines, and extracellular vesicles (88, 89). Mechanistically, this propagation can be mediated by both soluble SASP factors and extracellular vesicles (EVs) carrying proteins, lipids, and regulatory nucleic acids. In recipient cells, mediators including TGF-*β*-family ligands, VEGF, CCL2, CCL20, and IL-1-dependent signals can activate p15/p21- and p16/Rb-associated growth-arrest programs, thereby inducing secondary senescence and a local SASP that amplifies the initial signal. Experimental evidence supports both local and systemic propagation: SASP-mediated paracrine senescence has been demonstrated *in vitro* and *in vivo* (52), whereas EVs derived from aged bone marrow macrophages induced senescence-associated changes across multiple distant tissues in mice (90). However, these models do not establish how tumor- or therapy-derived SASP mediators are distributed among organs in BC.

Importantly, current evidence does not demonstrate selective cardiac targeting of circulating SASP factors or senescence-associated extracellular vesicles. Their tissue-specific effects are likely determined by the interaction between systemic exposure and local susceptibility, including vascular permeability, receptor expression, cellular composition, pre-existing tissue injury, and regenerative capacity. The apparent vulnerability of the cardiovascular system may therefore reflect its continuous exposure to circulating mediators, dense microvascular network, high mitochondrial and oxidative demands, and the limited regenerative capacity of adult cardiomyocytes. Moreover, direct injury from anthracyclines, radiotherapy, or HER2-targeted therapies may prime cardiac endothelial cells, cardiomyocytes, and fibroblasts, allowing circulating SASP signals to act as a second hit that initiates secondary senescence and local inflammatory amplification. Nevertheless, comparative multi-organ evidence establishing the heart as the most sensitive distant organ is currently lacking, and senescence propagation to the kidney, liver, lung, brain, and other tissues may be underrecognized. Longitudinal biodistribution and spatial multi-organ studies are therefore required to determine whether circulating SASP components exhibit genuine organotropism or act predominantly upon tissues rendered susceptible by treatment exposure and pre-existing vulnerability.

A key feature of SASP biology is its ability to dynamically reshape immune responses, reflecting a central, dual role for the immune system in both clearing and propagating therapy-induced senescent cells. In the early phases, SASP factors may support antitumor immunity by recruiting and activating immune cells that recognize and clear senescent tumor cells, limiting their excessive accumulation and preserving to tissue homeostasis (8). However, persistent SASP exposure may progressively impair this immune surveillance, promoting immunosenescence, immune exhaustion, and sustained inflammatory activation (8, 91–93).

Consistent with this, TIS cancer cells may progressively shift from an initially immunogenic state toward immune evasion and impaired immune-cell functionality, highlighting the context-dependent role of senescence in shaping long-term immune responses (61, 94). Supporting this immune-evasive phenotype, experimental evidence demonstrated that therapy-induced senescent cancer cells upregulate PD-L1 through ribophorin 1-dependent transcriptional and glycosylation mechanisms, thereby reducing their susceptibility to cytotoxic T-cell-mediated clearance (95). This progressive shift establishes a self-reinforcing cycle of inflammaging, tissue dysfunction, and senescence propagation across multiple organs and age-related comorbidities in cancer survivors (8, 26), while also amplifying immune–vascular crosstalk that sustains endothelial dysfunction, vascular inflammation, and chronic senescence propagation within systemic cardiovascular networks (63).

Both innate and adaptive immune populations — including NK cells, macrophages, neutrophils, dendritic cells, T cells, and B cells — participate in senescent cell recognition and clearance, although this surveillance progressively declines with aging, chronic inflammation, and persistent therapy-induced stress (93, 96–100). Crucially, no empirically validated temporal or quantitative threshold currently distinguishes effective early immune-mediated clearance of senescent cells from the subsequent development of chronic immune dysfunction, and this transition may represent a continuum rather than a discrete biological switch. Longitudinal studies in BC illustrate heterogeneous inflammatory trajectories. In 51 postmenopausal women with early BC, adjuvant chemotherapy induced a transient increase in circulating cytokines, which returned close to pre-treatment levels within six months (78). Conversely, in a cohort of 192 patients with early-stage BC, TNF-α, soluble TNF receptor II, IL-6, IFN-*γ*, and IL-8 remained elevated for up to 18 months among patients receiving combined chemotherapy and radiotherapy (79). However, neither study assessed senescent-cell burden, NK-cell cytotoxicity, macrophage-mediated clearance or polarization, or concurrent cardiovascular outcomes. Therefore, these data cannot explain why inflammation resolves in some patients but persists and contributes to long-term cardiovascular injury in others. This divergence may be influenced by senescent-cell burden and persistence, SASP composition, treatment intensity, age, baseline immune competence, and cardiometabolic vulnerability. Resolving this question will require prospective studies combining serial senescence biomarkers, immune-cell functional profiling, and longitudinal cardiovascular assessment.

In BC survivors, a progressive shift from surveillance toward chronic immune dysfunction may be particularly relevant because anticancer therapies, aging, and pre-existing cardiovascular risk factors can converge on a chronic inflammatory state resembling inflammaging. This inflammaging-like state is supported by persistent elevations in circulating biomarkers such as high-sensitivity (hs)-CRP and IL-6 (93, 101, 102), alongside immunophenotypic hallmarks of immunosenescence — including expansion of CD28-null and EMRA T-cell subsets and mitochondrial dysfunction within CD8+ compartments (91, 92). In this context, persistent TIS may contribute not only to local tumor or cardiovascular remodeling, but also to systemic immune-metabolic alterations that increase long-term vulnerability to both cancer recurrence and cardiovascular disease (26).

An additional emerging layer is represented by clonal hematopoiesis of indeterminate potential (CHIP), an age-related expansion of mutant hematopoietic clones carrying somatic mutations that is associated with chronic inflammation and cardiovascular risk. Cancer therapy may not merely coexist with CHIP but actively reshape clonal fitness. Longitudinal sequencing studies have shown that radiotherapy, platinum compounds, and topoisomerase II inhibitors preferentially select clones carrying DNA damage-response mutations, including TP53, PPM1D, and CHEK2 (103). More recently, analyses of serial samples from four randomized clinical trials confirmed chemotherapy-related expansion of TP53-mutant and other DNA damage-response clones, an effect attenuated by transient CDK4/6 inhibition (104). These findings support treatment-driven clonal selection, although they do not establish a direct mechanistic relationship with TIS.

Conversely, CHIP may amplify cardiovascular vulnerability following cancer therapy through pro-inflammatory myeloid signaling. In a cohort of 8,004 patients with solid tumors, CHIP was associated with increased heart failure risk after cancer therapy, with an exploratory interaction between CHIP and intensive chemotherapy (105). More specifically, in two independent BC cohorts, CHIP was associated with increased susceptibility to trastuzumab-related cardiotoxicity, particularly in patients carrying larger clones; Tet2-deficient clonal hematopoiesis also exacerbated trastuzumab-induced left ventricular dysfunction in mice (106). However, these studies did not assess TIS or longitudinal senescence biomarkers and therefore cannot determine whether CHIP promotes cardiovascular senescence, whether therapy-induced senescence facilitates clonal expansion, or whether the two processes form a self-reinforcing inflammatory circuit. Prospective BC cohorts combining pre- and post-treatment clonal sequencing with senescence biomarkers and longitudinal cardiac and vascular phenotyping are required to resolve this proposed bidirectional relationship. Collectively, these observations support the concept that SASP-driven immune remodeling and inflammaging represent key mechanisms through which TIS may propagate systemic dysfunction across tumor, cardiovascular, and aging-associated pathways. Persistent inflammatory signaling, defective immune surveillance, clonal hematopoietic alterations, and chronic senescence propagation may therefore contribute to the long-term coexistence of cancer progression, cardiovascular disease, frailty, and accelerated biological aging in BC survivors (4).

### 5.2 Senescence as a systemic bridge between cancer, cardiovascular disease and aging

Senescence provides a unifying biological framework through which cancer therapy, cardiovascular disease, and aging can be interpreted as interconnected rather than independent processes. Emerging evidence suggests that TIS may act as a systemic biological process capable of simultaneously influencing tumor progression, cardiovascular dysfunction, immune remodeling, and accelerated aging (2). In this perspective, TIS represents not only a local response to treatment-induced stress, but also a broader remodeling process that may affect multiple tissues over time.

Traditionally, cardiovascular complications following anticancer therapies have largely been interpreted as direct toxic effects of chemotherapy radiotherapy, or targeted agents. However, emerging evidence supports a broader paradigm in which persistent senescence-associated remodeling contributes to long-term cardiovascular vulnerability well beyond the acute treatment phase. Anticancer therapies induce senescence not only in tumor cells, where it initially contributes to tumor growth arrest and immune-mediated clearance, but also in ECs, cardiomyocytes, fibroblasts, VSMCs, immune cells, and stromal compartments. This systemic senescence burden may alter tissue homeostasis through chronic inflammation, impaired repair capacity, metabolic dysfunction, and persistent SASP-mediated intercellular communication across multiple organs (2, 5, 63).

From this perspective, TIS acts as a mediator of bidirectional crosstalk between the tumor ecosystem and the cardiovascular system. Within tumors, persistent senescence-associated signaling may contribute to immune escape, stromal activation, therapy resistance, and disease recurrence. In parallel, similar senescence-associated programs within cardiovascular tissues may promote progressive cardiovascular vulnerability and chronic tissue dysfunction. Thus, the same mechanisms that may initially contribute to antitumor efficacy can paradoxically generate long-term systemic vulnerability and cardiovascular impairment when senescent cells persist and immune-mediated clearance becomes ineffective.

This vulnerability may be further amplified in elderly individuals and patients with pre-existing cardiovascular or cardiometabolic risk factors, who frequently exhibit increased basal senescent cell burden, chronic low-grade inflammation, impaired immune surveillance and vascular dysfunction. Exposure to anticancer therapies may accelerate these pre-existing aging-associated processes, shifting vulnerable patients toward a phenotype of premature biological aging characterized by frailty, vascular stiffness, metabolic dysfunction, impaired tissue resilience, and increased cardiovascular susceptibility (4, 26).

From a cardio-oncology perspective, this framework has important translational implications. Cardiovascular disease in cancer survivors should not be interpreted solely as a consequence of cumulative treatment toxicity, but also as the result of chronic senescence-associated tissue remodeling immune–vascular dysfunction and therapy-related premature biological aging. This view may help to explain delayed cardiovascular emergence of clinical phenotypes increasingly recognized in cancer survivors, including HFpEF-like syndromes, accelerated atherosclerosis, microvascular dysfunction, vascular stiffness, hypertension, and thrombotic complications (1, 4, 5, 24, 26, 75).

Collectively, these observations position TIS at the intersection of cancer biology, cardiovascular disease, and aging, supporting its emerging role as a systemic biological mediator of long-term survivorship vulnerability. This perspective highlights the need for integrated cardio-oncology approaches aimed at identifying patients with increased senescence burden, defining biomarkers of therapy-related biological aging and developing strategies capable of preserving oncologic efficacy while limiting long-term cardiovascular dysfunction and premature biological aging.

### 5.3 Heterogeneity of TIS across breast cancer molecular subtypes: implications for precision cardio-oncology

The biological consequences and therapeutic relevance of TIS are unlikely to be uniform across BC subtypes. Rather, they reflect the interaction between intrinsic tumor features — including hormone receptor and HER2 signaling, TP53 status, apoptotic dependencies, and immune context — and the subtype-specific treatments responsible for inducing cellular stress. These differences may influence the establishment, persistence, and composition of the senescent phenotype, as well as the response to senescence-targeting interventions. However, current research largely discusses TIS mechanisms, cardiovascular consequences, and therapeutic strategies without subtype-stratified analysis, while direct comparisons across HR-positive/HER2-negative, HER2-positive, and TNBC/basal-like disease remain limited. This evidence gap constrains the translational applicability of current findings within a precision cardio-oncology framework, in which both cardiovascular risk and senescence-targeted interventions may need to be tailored to BC subtype.

In HR-positive/HER2-negative BC, the treatment context is dominated by endocrine therapy and, increasingly, CDK4/6 inhibitors, although chemotherapy remains relevant in higher-risk disease. CDK4/6 inhibition can induce a senescence-like arrest in tumor cells, but the associated secretory phenotype appears to depend on cellular and receptor context. For example, palbociclib induced senescence-associated features in both ER-positive MCF-7 and triple-negative MDA-MB-231 cells, whereas the magnitude and composition of the inflammatory secretome differed substantially between the two models, with greater IL-6 and IL-8 production in MDA-MB-231 cells (107). In postmenopausal patients, these tumor- and treatment-specific effects intersect with metabolic and vascular vulnerability, creating a distinct cardio-oncological context. Metformin has therefore attracted interest as a potential senomorphic intervention; however, discordant preclinical and clinical findings in postmenopausal HR-positive BC preclude conclusions regarding whether it favorably or adversely modulates TIS in this population, as discussed in Sec. 6.3.

In HER2-positive BC, TIS must be considered within a treatment landscape that includes anthracyclines, HER2-targeted antibodies, tyrosine kinase inhibitors, and antibody–drug conjugates, each with distinct tumor and cardiovascular effects. Recent preclinical evidence indicates that trastuzumab deruxtecan and its deruxtecan payload can induce sustained senescence-associated phenotypes in HER2-positive BC models, including persistent growth arrest, oxidative stress, metabolic reprogramming, and SASP production (108). However, the cardiovascular consequences of this response were not evaluated. Conversely, cardiac injury associated with anthracyclines and HER2-directed therapies has been extensively characterized, but its relationship with persistent cardiovascular senescence remains insufficiently defined. Consequently, whether senescence-targeting interventions can attenuate therapy-related cardiovascular injury in HER2-positive BC without compromising anticancer efficacy, tumor-cell clearance, or adaptive cardiac repair remains unknown, as their therapeutic potential and cardiovascular safety have not yet undergone dedicated validation in this subtype.

TNBC/basal-like BC presents a different biological setting, characterized by greater genomic instability, frequent TP53 alterations, a distinct immune microenvironment, and extensive exposure to senescence-inducing treatments such as anthracyclines and DNA-damaging agents. Emerging evidence indicates that senolytic sensitivity is heterogeneous even within TNBC. In a comparative study using the murine 4T1 and E0771 models, the effects of sequential doxorubicin and BCL-2-family inhibition differed according to both the senolytic agent and tumor model: Navitoclax showed activity in both models, whereas Venetoclax-mediated senolysis was evident primarily in E0771 cells (109). *In vivo* antitumor activity was subsequently demonstrated in the E0771 model, but cardiovascular function and senescence were not assessed. TP53 may represent an additional source of heterogeneity: earlier evidence indicated preferential elimination of chemotherapy-induced senescent cells by BH3 mimetics in TP53 wild-type BC (110), whereas a more recent study reported synergy between doxorubicin and Venetoclax across TP53 wild-type, mutant, and knockdown TNBC models (111). Thus, the predictive value of TP53 status remains unresolved rather than established. Sequential senogenic-senolytic strategies involving Navitoclax are discussed further in Sec. 6.2, but their ability to preserve cardiovascular function while improving tumor control has not yet been tested in integrated TNBC-bearing models.

Collectively, these findings support a subtype-informed rather than uniform approach to TIS. Future precision cardio-oncology studies should integrate BC subtype and genotype, treatment exposure, menopausal and metabolic status, immune context, baseline cardiovascular vulnerability, and tissue-specific senescence burden. Models assessing tumor response and recurrence together with cardiac and vascular function will be essential to determine whether senescence-targeting interventions can achieve a favorable therapeutic balance within each BC subtype.

## 6 Senescence-targeting strategies in cardio-oncology

### 6.1 Rationale for senescence-targeted interventions in cardio-oncology

TIS represents an attractive but challenging and biologically complex therapeutic target in cardio-oncology. On one hand, senescence contributes to the antitumor efficacy of several cancer therapies by enforcing proliferative arrest in damaged malignant cells and facilitating immune-mediated tumor control. On the other hand, the persistence of therapy-induced senescent cells in non-malignant tissues, including the cardiovascular system, may promote chronic inflammation, tissue remodeling, endothelial dysfunction, myocardial fibrosis, and accelerated cardiovascular aging (1, 3). Thus, selective modulation of persistent pathological senescence may offer a strategy to reduce long-term cardiovascular toxicity while preserving the oncologic benefits of treatment.

In BC survivors, this concept is particularly relevant because many patients are exposed to multimodal therapies with well-established cardiovascular effects, including anthracyclines, radiotherapy, HER2-targeted therapies, endocrine therapy, and, in selected contexts, targeted agents. Accordingly, senescence is increasingly viewed not as a passive by-product of therapy, but rather as a dynamic and potentially targetable biological process in cardio-oncology. However, senescence-targeting strategies cannot be considered broadly cardioprotective without careful biological and clinical contextualization. Since senescence may exert both protective and detrimental effects depending on timing, tissue compartment, and disease stage, treatment exposure, and immune competence, indiscriminate suppression or elimination of senescent cells could theoretically interfere with antitumor efficacy, immune surveillance, wound healing or tissue repair. Therefore, the therapeutic goal in cardio-oncology should not be the complete abolition of senescence, but rather the selective targeting of persistent, pathological senescent cell populations and their deleterious secretory activity. This requires a precise understanding of when senescence is adaptive and tumor-suppressive, and when it becomes chronic, maladaptive, and tissue-damaging. In practical terms, senescence-targeted interventions will likely need to be timed and tailored according to treatment phase, cancer status, cardiovascular risk profile, biological age, inflammatory burden, and senescence-associated biomarkers, thereby aligning with the emerging paradigm of precision cardio-oncology (112).

This perspective has led to growing interest in senotherapies, broadly divided into senolytics, which selectively eliminate senescent cells, and senomorphics, which attenuate harmful SASP signaling and chronic inflammatory remodeling without necessarily inducing senescent cell death (1, 32). Although these strategies remain largely preclinical in the context of cardio-oncology, they may represent a promising therapeutic avenue to mitigate long-term cardiovascular dysfunction, therapy-associated premature aging, and survivorship vulnerability while preserving the survival benefits of anticancer treatment.

### 6.2 Senolytic strategies: selective elimination of senescent cells

Senolytics represent a promising class of therapeutic agents designed to selectively eliminate senescent cells by targeting the pro-survival pathways that allow their persistence within tissues. By interfering with senescent cell anti-apoptotic pathways, these compounds preferentially induce apoptosis in senescent rather than proliferating cells (32, 113), thereby reducing senescent cell burden and attenuating chronic tissue remodeling (32). In cardio-oncology, this concept is particularly attractive because persistent senescent cells may contribute to chronic endothelial dysfunction, vascular remodeling, myocardial fibrosis, and therapy-associated premature cardiovascular aging. However, the therapeutic window for senolytic intervention remains uncertain, as senescent cells may also participate in tissue repair, immune surveillance, and tumor-suppressive responses.

Among the most extensively investigated senolytic approaches is the combination of Dasatinib and Quercetin (D + Q). Dasatinib, a broad-spectrum tyrosine kinase inhibitor, and Quercetin, a natural flavonoid with antioxidant and anti-inflammatory properties, exert complementary senolytic effects through targeting of senescence-associated pro-survival pathways. In preclinical models, D + Q reduced senescent cell burden — as assessed by decreased SA-*β*-gal activity, p16/p21/p53 expression, and SASP-associated inflammatory mediators — attenuated inflammatory remodeling, and improved vascular and tissue function, including reduced fibrosis and preserved cardiac function in aging models (1, 114–117). Notably, quercetin also exerts senolysis-independent vasoprotective effects, including eNOS activation and enhanced nitric oxide bioavailability (118), suggesting that its combination with dasatinib may further enhance cardiovascular protection thought complementary anti-apoptotic and antioxidant mechanisms (117). These observations are particularly relevant in cardio-oncology, where endothelial dysfunction and chronic vascular remodeling represent major components of therapy-related cardiovascular toxicity. However, these benefits have largely been demonstrated in aging or non-oncological cardiovascular models rather than in BC-bearing models exposed to cardiotoxic anticancer therapy. Moreover, senolytic efficacy remains highly context-dependent, and current compounds still lack optimal selectivity together with robust cardiovascular clinical validation and long-term safety data in cancer survivors (115, 119). A further translational uncertainty concerns the long-term immunological safety of D + Q. Available clinical evidence derives predominantly from the continuous administration of Dasatinib at oncological doses in patients with hematological malignancies rather than from the intermittent schedules employed for senolytic purposes. In a nationwide register-based cohort study, patients with chronic myeloid leukemia receiving TKI therapy had a higher incidence of pneumonia than matched population controls, while within-cohort comparisons showed an increased incidence of acute upper respiratory infections among Dasatinib-treated patients compared with those receiving Imatinib (120). These findings raise the possibility that prolonged Dasatinib exposure may increase susceptibility to infectious complications. Conversely, other clinical and experimental studies have documented Dasatinib-associated mobilization and expansion of cytotoxic lymphocytes, including large granular lymphocyte, T-cell, and NK-cell populations, in some cases correlating with favorable treatment responses (121). More recent experimental evidence indicates that Dasatinib can also promote *γδ* T-cell expansion and memory-associated effector phenotypes, with enhanced antitumor activity against TNBC cells (122). The immune effect of Dasatinib therefore appears immunomodulatory rather than uniformly immunosuppressive and may depend on dose, treatment duration, underlying disease, and immune status. Whether intermittent D + Q regimens reproduce either the increased susceptibility to infection or the lymphocyte-expansion phenotype observed in these distinct clinical and experimental settings remains unknown, particularly in BC survivors. This lack of BC-specific evidence represents an additional translational uncertainty for D + Q-based senolytic strategies.

Another widely studied senolytic agent is Navitoclax (ABT-263), an orally bioavailable inhibitor of anti-apoptotic BCL-2 family proteins, including BCL-2, BCL-XL, and BCL-W. By disrupting mitochondrial survival signaling, Navitoclax selectively induces apoptosis in senescent cells dependent on BCL-2 family-mediated pathways for survival (32). In a non-tumor-bearing mouse model of doxorubicin-induced cardiotoxicity, Navitoclax reduced cardiac senescence and cardiotoxicity markers and restored cardiac function, as assessed longitudinally by echocardiography (86). Although these findings provide direct evidence that senolytic clearance may attenuate anthracycline-related cardiac injury, the absence of a concomitant tumor prevented assessment of whether Navitoclax affected the antitumor efficacy of doxorubicin. In a distinct model of age-related neurovascular dysfunction, Navitoclax significantly reduced both the number and total volume of spontaneous cerebral microhemorrhages in aged hypertensive mice, consistent with improved neurovascular integrity (119). Despite these promising findings, clinical translation remains limited by dose-dependent toxicities, particularly thrombocytopenia associated with inhibition of BCL-XL, an essential survival protein in mature platelets (123, 124). Because this toxicity is an on-target pharmacological effect rather than nonspecific off-target injury, it has historically constrained the dose intensity and clinical development of Navitoclax-based regimens. To address this bottleneck, platelet-sparing alternatives are under investigation. DT2216 is a BCL-XL-targeting proteolysis-targeting chimera (PROTAC) designed to recruit the von Hippel–Lindau E3 ligase, which is minimally expressed in platelets, thereby promoting BCL-XL degradation preferentially in nucleated cells. In a first-in-human phase I study involving patients with advanced solid tumors, DT2216 produced rapid and sustained BCL-XL degradation in peripheral leukocytes and predominantly transient thrombocytopenia without major bleeding events. Nevertheless, thrombocytopenia remained the most frequent treatment-related adverse event, and the study assessed DT2216 as an antitumor agent rather than as a senolytic intervention (125). An alternative preclinical strategy involves galacto-conjugated Navitoclax prodrug designed for preferential activation in senescence-associated *β*-galactosidase-positive cells. This approach increased senolytic specificity and reduced platelet toxicity relative to unconjugated Navitoclax in experimental models (126). Although these approaches may widen the therapeutic window of BCL-XL-directed interventions, their efficacy as senolytics and their long-term cardiovascular and oncological safety have not been established in BC-bearing models or BC survivors. Clinical studies of Navitoclax-based regimens, including the phase II REFINE trial (NCT03222609) in myelofibrosis, provide regarding the pharmacological feasibility and toxicity in humans, but were not designed to evaluate senescent-cell clearance, cardiovascular protection or preservation of antitumor efficacy in BC (127). Their relevance to cardio-oncology and BC survivorship therefore remains uncertain.

Fisetin, a naturally occurring flavonoid with antioxidant and anti-inflammatory properties, has also emerged as a promising senotherapeutic candidate. Rather than targeting a single pathway, fisetin broadly modulates senescence-associated signaling networks, including suppression of NF-*κ*B activity and reduction of SASP mediators such as IL-6 and TNF-α (1). Preclinical studies demonstrated that fisetin reduces senescence cell and attenuates inflammatory signaling in multiple tissues while displaying a relatively favorable safety profile (128, 129). These effects have been demonstrated across a broad translational spectrum — including senescent murine and human fibroblasts *in vitro*, progeroid (Ercc1−/Δ) and aged wild-type mice *in vivo,* and human adipose tissue explants — with significant, although cell type-specific and incomplete, reductions in senescence and SASP-associated markers across adipose tissue, spleen, liver, and kidney (128). Functionally, intermittent fisetin supplementation has been shown to improve endothelial function and reduce arterial stiffness in aged mice by decreasing endothelial cell senescence and enhancing NO bioavailability (130). These findings provide direct evidence of organ-level vascular benefit but derive from aging rather than BC or anticancer therapy-related cardiovascular models. Although fisetin exhibits relatively weak and context-dependent senolytic activity (131, 132), its translational relevance to BC survivorship is being investigated in ongoing phase II clinical trials evaluating frailty and physical function following chemotherapy exposure (1, 133, 134).

Taken together, the available evidence remains markedly compartmentalized, because the antitumor efficacy and cardiovascular effects of senolytic strategies have generally been evaluated in separate experimental models. As discussed above, Navitoclax reduced cardiac senescence burden and improved cardiac function in a non-tumor-bearing model of doxorubicin-induced cardiotoxicity, but its effects on tumor control could not be assessed (86). Conversely, BC-specific studies have evaluated senescent tumor-cell clearance and tumor control without concurrent cardiovascular assessment. In an orthotopic MDA-MB-231 xenograft model established in immunodeficient mice, sequential administration of doxorubicin followed by ABT-263 selectively targeted therapy-induced senescent tumor cells and resulted in marked and prolonged tumor suppression (135). In an orthotopic immunocompetent model of triple-negative BC, palbociclib-induced senescence followed by nano-encapsulated Navitoclax inhibited tumor growth and metastatic dissemination while reducing the systemic toxicity associated with Navitoclax (136). However, neither study included cardiac functional or cardiovascular senescence endpoints. A recent systematic review of 36 murine studies similarly supported the antitumor efficacy of sequential senogenic-senolytic strategies across breast, colorectal, ovarian, and other cancer models, while identifying substantial heterogeneity in experimental design and outcome reporting and a lack of concurrent cardiovascular functional assessment (137). Therefore, it remains unknown whether senolytic clearance can protect the cardiovascular system while preserving—or potentially enhancing—the oncological efficacy of senescence-inducing therapies in BC. Integrated BC-bearing models simultaneously assessing tumor response and recurrence, cardiac and vascular function, and tissue-specific or systemic senescence burden are urgently required to resolve this bidirectional trade-off.

Overall, senolytic efficacy remains highly context-dependent, and currently available compounds still lack optimal cell-type and tissue specificity, robust cardiovascular clinical validation, and long-term safety data in cancer survivors (115, 119). Despite encouraging preclinical and early translational findings, the clinical implementation of senolytic therapies in cardio-oncology remains challenging. The long-term impact of senolytic interventions on tissue repair, immune surveillance, and antitumor efficacy remains incompletely understood, particularly in BC survivorship settings characterized by chronic cardiovascular vulnerability. Further studies will be required to define optimal treatment timing, dosing schedule, patient selection, biomarker-guided monitoring and long-term cardiovascular safety before senolytic therapies can be integrated into routine cardio-oncology care. In parallel, robust approaches for quantifying the magnitude and spatial distribution of senescent cell burden across tumor and cardiovascular tissues will be essential to optimize patient selection and longitudinal monitoring of senolytic therapies, as discussed in the following section.

### 6.3 Senomorphic and anti-SASP approaches

An alternative and potentially complementary strategy is represented by senomorphics, a class of agents that attenuate the deleterious effects of senescent cells without necessarily inducing their elimination. Unlike senolytics, senomorphics primarily target SASP-associated inflammatory, metabolic, and stress-response pathways, thereby reducing chronic inflammatory remodeling and tissue dysfunction while potentially preserving the beneficial short-term effects of senescence, including tissue repair, immune surveillance and antitumor growth arrest (1). This distinction may be particularly relevant in cardio-oncology, where complete elimination of senescent cells could theoretically interfere with adaptive tissue responses or antitumor efficacy, whereas modulation of chronic SASP signaling may attenuate long-term cardiovascular injury with a potentially more favorable safety profile.

Several signaling pathways implicated in SASP regulation have emerged as attractive therapeutic targets, including mTOR, NF-*κ*B, p38 MAPK, AMPK, and JAK/STAT signaling. Among currently investigated senomorphic agents, metformin, rapamycin, and JAK/IL-6 pathway inhibitors are particularly relevant in cardio-oncology because they target mechanisms strongly implicated in therapy-induced cardiovascular senescence and chronic inflammatory remodeling.

Metformin, widely used in the treatment of type 2 diabetes mellitus, exerts senomorphic activity primarily through AMPK activation, downstream mTOR inhibition, and suppression of NF-*κ*B-associated pro-inflammatory signaling.

These effects are associated with attenuation of oxidative stress, suppression of SASP-associated inflammatory signaling, and preservation of endothelial homeostasis (1, 138). In experimental cardiovascular models exposed to anticancer therapies, metformin reduced vascular inflammation and endothelial dysfunction, supporting its potential role in limiting therapy-induced vascular damage. In human aging-related clinical studies, metformin has been explored as a candidate geroprotective intervention. The completed Metformin in Longevity Study (MILES), a small double-blind, placebo-controlled crossover trial, demonstrated significant metformin-associated transcriptional changes in skeletal muscle (647 genes) and subcutaneous adipose tissue (146 genes) of older adults with impaired glucose tolerance, including modulation of several aging-associated pathways. Although these findings require validation in larger cohorts and do not establish metformin's primary tissue of action, they provide proof-of-concept for the biological effects of metformin on aging-associated pathways (1, 139–142). The large Targeting Aging with Metformin (TAME) 6-year trial was designed to evaluate whether metformin can delay age-related multimorbidity (1, 143); however, efficacy results are not yet available. However, definitive evidence that metformin directly reduces senescence burden or prevents therapy-induced cardiovascular senescence in humans remains limited. A further unresolved bidirectionality emerges in postmenopausal HR-positive BC. Preclinical models suggest that metformin may inhibit stromal aromatase expression and reduce tumor progression in postmenopausal ER-positive mammary tumors (144). Conversely, a randomized phase II trial combining metformin with an aromatase inhibitor in postmenopausal patients with HR-positive locally advanced or metastatic BC showed a non-significant trend toward shorter progression-free survival in the metformin arm compared with placebo (4.7 vs. 6.0 months; HR, 1.2) (145). Although this finding does not establish a detrimental effect of metformin, it contrasts with the anticipated benefit derived from preclinical evidence. Moreover, neither study directly assessed therapy-induced senescence, preventing conclusions as to whether metformin favorably or adversely modulates TIS in this clinical setting. This discordance underscores that the interactions among metformin, hormone receptor signaling, and senescence pathways remain incompletely understood and warrants caution before incorporating metformin into senomorphic strategies for postmenopausal survivors of HR-positive BC. Therefore, metformin should currently be regarded as potential senomorphic candidate in cardio-oncology, but its clinical utility remains to be established.

Beyond their established cardiometabolic activity, emerging evidence suggests that sodium-glucose cotransporter-2 (SGLT2) inhibitors may also modulate senescence through mechanisms involving immune-mediated senolysis. Experimental studies demonstrated that canagliflozin may enhance immune-mediated clearance of senescent cells through downregulation of PD-L1 expression, thereby restoring senescence surveillance and attenuating chronic inflammatory remodeling (49, 146). This concept is biologically supported by evidence that senescent cells may exploit PD-L1/PD-1 immune checkpoint signaling to evade immune-mediated clearance, thereby contributing to persistence of senescent cell burden and chronic tissue dysfunction (49). These observations, together with evidence that modulation of SGLT signaling attenuates endothelial senescence through redox-sensitive mechanisms (27) further support the broader concept that targeting immune–metabolic pathways may represent a promising strategy to limit therapy-associated cardiovascular aging and dysfunction. Similarly, glucagon-like peptide-1 (GLP-1)-associated signaling has been implicated in attenuation of endothelial senescence and oxidative stress following exposure to antimetabolites such as 5-fluorouracil, further supporting the potential relevance of cardiometabolic modulation in therapy-associated vascular aging (70). However, these findings derive predominantly from non-oncological aging models and *in vitro* endothelial systems. Whether SGLT2 inhibition or GLP-1 receptor modulation prevents therapy-induced cardiovascular senescence while preserving antitumor efficacy has not been evaluated in BC-bearing models or BC survivors.

Rapamycin, a direct inhibitor of mTOR signaling, has also demonstrated substantial senomorphic potential through attenuation of SASP-associated inflammatory activity and modulation of nutrient-sensing pathways involved in senescence maintenance. In preclinical models of chemotherapy-associated cardiovascular injury, mTOR inhibition reduced senescence markers, inflammatory activation, and maladaptive tissue remodeling, while preserving regenerative capacity and limiting myocardial dysfunction (63, 147–150). These observations suggest that rapamycin-based strategies may represent a promising approach to mitigate chronic cardiovascular remodeling associated with therapy-induced premature aging. However, early clinical evidence remains limited, and a small clinical trial did not conclusively demonstrate significant effects of rapamycin on senescence markers or age-associated phenotypes (1, 150). Similarly, resveratrol, a naturally occurring polyphenol with antioxidant, anti-inflammatory, and SIRT1-activating properties, has emerged as another promising senomorphic candidate. Experimental studies have shown that resveratrol attenuates myocardial senescence, suppresses NLRP3 inflammasome activation, and reduces oxidative stress–driven inflammatory signaling following ischemic injury, while preserving cardiac function (151). In addition, resveratrol protects against doxorubicin-induced cardiotoxicity in aged hearts through activation of the SIRT1–USP7 signaling axis, thereby limiting therapy-associated cellular senescence and myocardial dysfunction (152). Although its clinical translation remains limited by low bioavailability and heterogeneous efficacy across studies, these findings further support modulation of oxidative stress and senescence-associated signaling as a promising strategy to mitigate therapy-induced cardiovascular remodeling.

Another important therapeutic target is the JAK/IL-6 inflammator*y* axis, which plays a central role in SASP amplification and chronic vascular inflammation. Inhibition of JAK/STAT signaling attenuates SASP-associated cytokine production and endothelial activation, leukocyte recruitment, and pro-thrombotic signaling. In this context, ruxolitinib, a JAK1/2 inhibitor, has shown the ability to reduce SASP-associated inflammatory output and improve endothelial function in preclinical cardiovascular models (153). However, as observed with other anti-inflammatory strategies, such as IL-1β inhibition in the CANTOS trial (154), modulation of inflammatory pathways may also compromise physiological immune responses. Consistently, ruxolitinib has been associated with an increased risk of opportunistic infections, reflecting its immunosuppressive effects on cytokine signaling and lymphocyte function (155, 156). This immune trade-off highlights the need to carefully balance cardiovascular benefit against immune-related adverse effects when developing senomorphic strategies for cardio-oncology. Nevertheless, preclinical evidence supports the concept that anti-inflammatory senomorphic strategies may attenuate chronic cardiovascular dysfunction without requiring complete elimination of senescent cells.

Emerging evidence further suggests that non-coding RNAs, including microRNAs (miRNAs) and long non-coding RNAs (lncRNAs), may represent an additional layer of senomorphic-associated regulation and a potential future therapeutic target (157). Several miRNAs implicated in cardiovascular aging and senescence, such as miR-34a, miR-17-3p, and miR-22, have been associated with mitochondrial dysfunction, fibrosis, endothelial senescence, and maladaptive cardiac remodeling through modulation of pathways involving SIRT1, p53, and oxidative stress signaling, and extracellular matrix regulation (158–160). In parallel, lncRNAs such as MALAT1 have been linked to regulation of senescence-associated gene expression and cardiac aging phenotypes (161). Although these approaches remain highly experimental, modulation of non-coding RNA networks may represent a promising future strategy for targeting therapy-induced cardiovascular senescence and inflammaging.

Compared with senolytics, senomorphic approaches may theoretically offer a more favorable safety profile in cardio-oncology by attenuating chronic inflammatory signaling while preserving beneficial transient senescence programs involved in tissue homeostasis and repair. However, prolonged suppression of SASP-associated pathways could also interfere with immune surveillance, senescent cell clearance, antitumor immunity and adaptive reparative responses (162). Crucially, it whether attenuation of chronic SASP signaling can mitigate therapy-induced cardiovascular injury without compromising tumor control remains unknown, as these outcomes have not been evaluated concurrently in BC-specific experimental models. Integrated studies assessing cardiac and vascular function alongside tumor response and recurrence are therefore required, together with optimization of treatment timing, duration, dose intensity and patient selection (Table 2).

**Table 2:** Translational senescence-targeting interventions in cardio-oncology.

Strategy	Representative agents	Senescence-associated molecular targets/pathways	Potential cardio-oncology applications	Main limitations/concerns	Translational status	Representative references
Senolytics	Dasatinib + Quercetin (D + Q)	Anti-apoptotic pathways; BCL-xL; PI3 K/AKT; SASP-associated signaling	Prevention of therapy-related endothelial dysfunction; reduction of myocardial remodeling and fibrosis; mitigation of inflammaging and CTR-CVA phenotypes	Limited cell-type specificity; potential off-target toxicity; variable efficacy across tissues	Strong preclinical evidence; early-phase clinical studies ongoing	(63, 114, 117, 185)
	Navitoclax (ABT-263)	BCL-2 family proteins (BCL-2, BCL-xL, BCL-w)	Reduction of senescent-cell burden; attenuation of vascular and myocardial remodeling; prevention of persistent senescence-associated dysfunction	On-target thrombocytopenia and hematologic toxicity; limited therapeutic window; platelet-sparing derivatives remain investigational	Strong preclinical evidence; limited clinical applicability	(63, 86, 119, 123–127, 186)
	Fisetin	PI3K/AKT; NF-*κ*B; oxidative stress pathways	Improvement of endothelial function; attenuation of vascular senescence and oxidative stress; potential prevention of vascular aging	Limited bioavailability; variable potency; incomplete clinical validation	Promising preclinical evidence; early clinical evaluation ongoing	(63, 128, 130, 133, 134, 187)
Senomorphics/anti-SASP agents	Metformin	AMPK activation; mTOR inhibition; NF-κB suppression; SASP modulation	Reduction of chronic inflammation and metabolic dysfunction; attenuation of cardiovascular aging burden in cancer survivors	Gastrointestinal side effects and renal limitations; context-dependent oncological effects; uncertain long-term efficacy	Clinically available repurposed agent; senomorphic efficacy in cardio-oncology remains unestablished	(63, 142, 144, 145, 188–190)
	Rapamycin *(e.g., everolimus)*	mTOR inhibition; autophagy activation; SASP suppression	Reduction of vascular aging, fibrosis, and maladaptive remodeling; preservation of cardiovascular function during survivorship	Immunosuppressive and metabolic side effects; narrow therapeutic window	Strong preclinical evidence; limited early clinical evidence in cardiovascular aging	(63, 147, 149, 150, 191)
	SGLT2 inhibitors *(e.g., canagliflozin)*	ROS/p38/JNK inhibition; ROS/ERK modulation; immune surveillance restoration; senescent-cell clearance; NF-κB suppression	Potential prevention of therapy-related HF phenotypes and vascular dysfunction; reduction of vascular inflammation and senescent-cell accumulation	Limited senescence-specific clinical evidence	Strong cardiovascular clinical evidence; senescence-specific translation remains preclinical	(27, 146, 192, 193)
	GLP-1 receptor agonists	cAMP/PKA; PI3K; ERK1/2 signaling	Improvement of endothelial function and cardiometabolic homeostasis; attenuation of endothelial senescence and inflammation	Long-term senescence-related effects remain under investigation	Established cardiometabolic strategy	(31, 63, 70)
	Resveratrol/polyphenols	SIRT1 activation; antioxidant signaling; NLRP3 suppression; anti-inflammatory pathways	Reduction of oxidative stress and NLRP3-mediated inflammation; attenuation of myocardial senescence; protection against anthracycline-induced cardiotoxicity; preservation of endothelial and cardiac function	Bioavailability limitations; heterogeneous clinical efficacy	Strong preclinical evidence; limited clinical translation	(63, 151, 152)
	JAK/IL-6 inhibitors *(e.g., ruxolitinib)*	JAK1/2–STAT3 inhibition; SASP suppression; attenuation of IL-6-driven inflammatory signaling	Potential reduction of chronic vascular inflammation, endothelial dysfunction, and SASP-associated cardiovascular remodeling; attenuation of therapy-induced cardiovascular senescence	Risk of immunosuppression and opportunistic infections; potential interference with physiological immune surveillance	Promising preclinical evidence; limited cardiovascular clinical validation	(153–156)
Immune-mediated senescence targeting	Anti-PD-L1 strategies; CAR-T approaches	Immune-mediated clearance of senescent cells; restoration of senescence surveillance	Enhancement of immune surveillance while limiting persistent senescent-cell accumulation; potential reduction of vascular senescence and chronic inflammation	Safety concerns; risk of excessive immune activation; limited tissue specificity	Early translational stage	(49, 63, 194)

### 6.4 Timing, patient selection, and preservation of antitumor efficacy

One of the major challenges in the clinical translation of senescence-targeting strategies is the need to preserve the beneficial antitumor effects of TIS while limiting its long-term deleterious consequences. Senescence is not intrinsically harmful; rather, its biological impact depends on timing, persistence, tissue context, disease stage and the balance between adaptive and pathological remodeling. Consequently, indiscriminate suppression or elimination of senescent cells may interfere with antitumor efficacy, tissue repair, or immune-mediated clearance mechanisms (3). In cardio-oncology, this duality is particularly relevant because therapeutic interventions should ideally attenuate persistent pathological senescence within cardiovascular and non-malignant tissues without compromising tumor control.

In this context, increasing attention has been directed toward the temporal optimization of senotherapies. Emerging evidence supports intermittent or delayed administration strategies, in which senolytic or senomorphic interventions are temporally separated from cytotoxic treatments in order to preserve the acute tumor-suppressive effects of senescence while targeting persistent senescent cell populations responsible for chronic tissue dysfunction (163). BC-specific support for this sequential principle is provided by experimental studies in which doxorubicin-induced senescence followed by ABT-263, or palbociclib-induced senescence followed by nano-encapsulated Navitoclax, improved tumor control, as discussed in Sec. 6.2 (135, 136). Intermittent senolytic administration, often described as a “hit-and-run” strategy, aims to periodically reduce senescent cell burden once senescence-associated remodeling becomes maladaptive, rather than continuously suppressing senescence-related pathways. This strategy is based on the biological rationale that senescent cells persist long after the initial therapeutic insult and therefore require only periodic clearance, allowing drug-free intervals that may minimize off-target toxicity while preserving beneficial transient senescence programs involved in tissue repair and physiological homeostasis, with treatment timing ideally informed by senescence biomarkers such as p16INK4a and SASP protein panels (163). This approach may be particularly attractive in survivorship settings, where the therapeutic objective is not to block the initial anticancer response, but to limit the long-term persistence of senescent cells and their deleterious SASP-driven remodeling.

Preclinical and early translational studies have provided support for this strategy. Intermittent administration of D + Q reduced senescent cell accumulation, attenuated chronic inflammation, improved physical function, and extended lifespan in models of natural aging and in patients with idiopathic pulmonary fibrosis, while limiting excessive systemic toxicity and preserving adaptive transient senescence responses involved in tissue homeostasis (164, 165). Although these studies were not performed in tumor-bearing models, emerging evidence in oncology supports the same timing-dependent principle. In a preclinical melanoma model, administration of D + Q seven days after senescence-inducing radiotherapy reduced tumor growth and prolonged survival, whereas immediate post-radiotherapy treatment failed to enhance antitumor efficacy, underscoring that the therapeutic benefit of senolytic interventions critically depends on their temporal separation from the initial cytotoxic insult (166). Conversely, prolonged or continuous senomorphic treatment may increase the risk of off-target effects, including impaired tissue repair, altered immune homeostasis, and interference with physiological senescence programs involved in wound healing and regeneration (167). Similarly, overdosing or prolonged exposure to senolytic agents may increase treatment-related toxicities, as exemplified by Navitoclax-associated thrombocytopenia due to BCL-XL inhibition (168). Thus, careful definition of treatment timing, dosing strategies, duration, and therapeutic window will likely represent a critical determinant of both therapeutic efficacy and safety. Realizing this potential will also require validated diagnostic and longitudinal monitoring approaches capable of accurately quantifying senescence burden and evaluating therapeutic response.

Patient selection represents another crucial aspect for the future integration of senescence-targeting approaches into cardio-oncology. The greatest therapeutic benefit may be expected in individuals at high risk of accumulating a substantial senescence cell burden that could drive therapy-associated cardiovascular dysfunction, including elderly patients, individuals with pre-existing cardiovascular disease or cardiometabolic risk factors, and BC survivors exposed to cumulative cardiotoxic therapies such as anthracyclines or thoracic radiotherapy (4, 138). Early identification of these high-risk populations, before irreversible cardiovascular remodeling becomes established, may maximize the effectiveness of senescence-targeting interventions and further reinforce the importance of individualized risk stratification throughout BC survivorship (169, 170).

Future integration of senescence-targeting strategies into cardio-oncology practice may additionally benefit from established cardiovascular risk assessment tools, particularly those recommended by the 2022 ESC Guidelines on cardio-oncology, developed in collaboration with the European Hematology Association, the European Society for Therapeutic Radiology and Oncology, and the International Cardio-Oncology Society (6, 171). These guidelines incorporate structured baseline cardiovascular risk stratification, including HFA-ICOS-based approaches, to identify patients at increased risk of cancer therapy-related cardiovascular toxicity before and during treatment. SCORE2-based cardiovascular risk estimation may additionally help contextualize long-term atherosclerotic and vascular risk, particularly in survivorship settings. Integration of these clinical tools with senescence-associated biomarker profiling could support a transition from generic cardioprotection toward precision cardio-oncology strategies tailored to biological age, inflammatory burden, treatment exposure, and individual cardiovascular risk.

In this perspective, biomarker-guided stratification may become essential for the implementation of precision senotherapy in cardio-oncology. Candidate biomarkers include expression of senescence-associated molecules (p16^INK4a^ and p21^CIP1^), circulating SASP mediators (IL-6, MCP-1, TNF-α), as well as markers of persistent DDR activation such as *γ*H2AX (7). Additional candidates encompass circulating inflammatory proteins, extracellular vesicle-associated signals, immune-aging signatures, mitochondrial dysfunction markers, and multi-omics profiles reflecting biological aging and therapy-associated tissue stress. However, none of these biomarkers is currently sufficiently validated to guide senotherapy in routine cardio-oncology practice, and their interpretation will require standardized assays, longitudinal validation, and integration with clinical endpoints. Emerging nanotechnology-based approaches may further improve detection and monitoring of vascular senescence in cardiovascular disease settings. For example, VCAM-1-targeted fluorescent nanocarriers have been proposed as a strategy to detect senescent ECs and vascular inflammation, potentially enabling more precise identification of senescence-associated vascular injury (172). Although still experimental, these approaches illustrate how molecular imaging and targeted nanodiagnostics may complement circulating biomarkers and conventional cardiovascular imaging in the future assessment of therapy-related vascular aging.

Realizing this potential, however, will require novel approaches capable of resolving not only the presence but also the spatial distribution and burden of senescent cells within cardiovascular tissues. Emerging single-cell and spatially resolved technologies — including spatial transcriptomics, multiplexed imaging, and deep learning–based tissue mapping (173) — together with novel PET radioligands targeting senescence-associated pathways, such as *β*-galactosidase — and fibroblast activation protein (FAP)-targeted tracers already explored in cardiac and other organ contexts (174) — may help bridge this gap, by enabling non-invasive, longitudinal characterization of tissue senescence, thereby improving patient stratification and guiding the timing and intensity of senescence-targeting interventions. Integration of biological aging markers with advanced molecular imaging, spatially resolved tissue profiling, circulating multi-omics, and functional assessment may further improve identification of patients at increased risk for therapy-related cardiovascular dysfunction. In parallel, emerging artificial intelligence (AI)-assisted risk prediction models and digital health approaches may support more precise longitudinal monitoring, early detection of subclinical cardiovascular decline, and individualized survivorship care (Figure 3).

**Figure 3:**
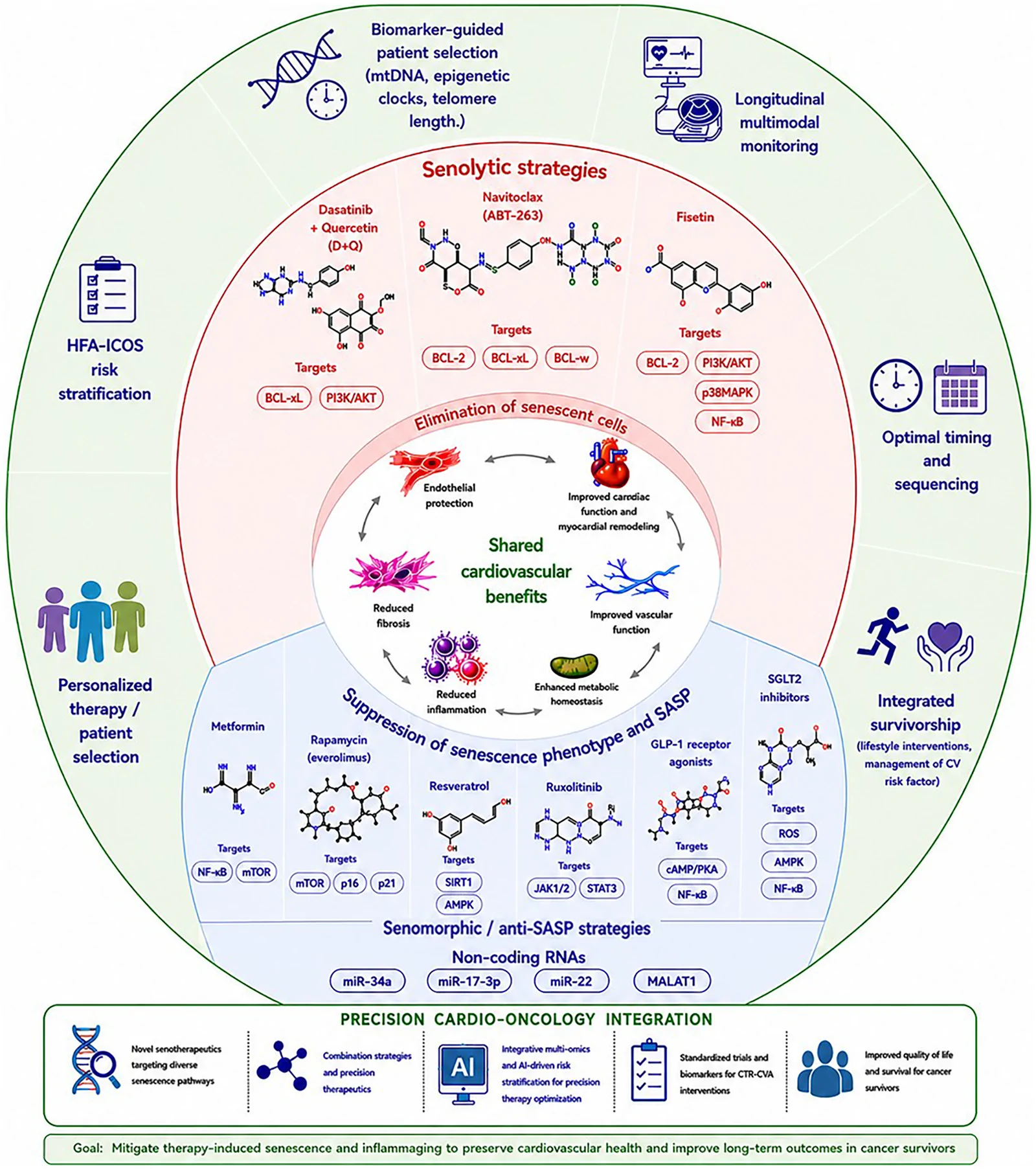
Senescence-targeting interventions and precision cardio-oncology strategies for the prevention of therapy-related cardiovascular aging. Senescence-targeting approaches in cardio-oncology can be broadly categorized into senolytic strategies, which selectively eliminate senescent cells, and senomorphic/anti-SASP strategies, which suppress deleterious senescence-associated signaling without inducing cell death. Despite targeting distinct molecular pathways, these strategies converge toward shared cardiovascular benefits, including improved endothelial and vascular function, preserved cardiac function and myocardial remodeling, reduced fibrosis and chronic inflammation, and enhanced metabolic homeostasis. The outer ring highlights the integration of senescence-targeting interventions into precision cardio-oncology frameworks aimed at mitigating therapy-induced senescence, reducing CTR-CVA, and improving long-term cardiovascular health in cancer survivors. *HFA-ICOS, Heart Failure Association–International Cardio-Oncology Society; CTR-CVA, cancer therapy-related cardiovascular aging*. FigureLabs Plus (v4.1), an AI-powered scientific illustration platform, was used to assist with the generation of the graphical elements of this figure. The scientific content and graphical design were verified, reviewed, and finalized by the authors. Created with FigureLabs (https://figurelabs.ai).

Ultimately, the clinical success of senescence-targeting interventions will depend on the ability to selectively target pathological persistent senescence while preserving transient senescence programs required for tumor control and tissue homeostasis. Achieving this balance represents one of the major translational challenges in the future development of precision cardio-oncology therapies.

### 6.5 Integration of senescence-targeting strategies into cardio-oncology care

The integration of senescence-targeting strategies into cardio-oncology care represents a promising but still largely exploratory therapeutic frontier. Given the contribution of TIS to chronic cardiovascular remodeling, vascular dysfunction, myocardial injury, and accelerated biological aging, senotherapies may offer an opportunity to move cardio-oncology beyond the conventional management of overt cardiotoxicity toward prevention of therapy-induced biological aging and long-term cardiovascular vulnerability.

In practical terms, senescence-targeting interventions should be considered within a precision cardio-oncology framework. Rather than being broadly administered to all patients receiving potentially cardiotoxic therapies, senescence-targeting approaches will likely require individualized risk stratification based on treatment exposure, cardiovascular risk profile, biological age, inflammatory status, immune competence, and senescence-associated biomarkers. BC survivors exposed to anthracyclines, left-sided thoracic radiotherapy, HER2-targeted therapies, or multimodal treatment combinations may represent a particularly relevant population for future studies, especially when additional risk modifiers such as older age, hypertension, diabetes, obesity, hypercholesterolemia or pre-existing cardiovascular disease are present.

A major unmet need is the development of integrated preclinical studies followed by dedicated clinical trials designed to determine whether senescence-targeting strategies can effectively reduce cardiovascular toxicity without compromising oncologic outcomes. Preclinical investigations should use immunocompetent BC-bearing models exposed to clinically relevant anthracycline, radiotherapy, HER2-targeted, or subtype-specific treatment regimens and should simultaneously assess primary tumor response, metastatic progression and recurrence; cardiac and vascular function, fibrosis, and thrombotic risk; and the temporal and tissue-specific burden of senescence and SASP activation. Subsequent trials should incorporate both cardiovascular and cancer-related endpoints, including cardiac function, vascular stiffness, circulating inflammatory and senescence-associated biomarkers, quality of life, physical performance, cancer recurrence, and treatment efficacy. Longitudinal follow-up will be essential, given that senescence-associated cardiovascular dysfunction may emerge years or even decades after treatment completion. In this context, ongoing prospective studies such as CARDIOCARE and CARTIER provide important examples of the evolving precision cardio-oncology paradigm (175, 176). Although not specifically designed to evaluate senescence-targeting therapies, they illustrate how biomarker-driven risk stratification, digital health monitoring, artificial intelligence-assisted prediction, and multidisciplinary cardiovascular surveillance can be integrated into personalized care for cancer patients and survivors (ClinicalTrials.gov: NCT03711110; NCT06334445). These studies may provide useful methodological frameworks for future trials aimed at incorporating biological aging assessment, senescence-associated biomarkers, and longitudinal cardiovascular phenotyping into survivorship care.

Despite encouraging preclinical evidence, several biological and translational challenges still limit the clinical implementation of senotherapies. Senescent cells exhibit marked heterogeneity across tissues and disease contexts, with substantial variability in SASP composition, metabolic adaptations, immune interactions, and dependence on pro-survival pathways (177, 178). This heterogeneity complicates the development of universal senescence-targeting approaches and suggests that senotherapies will likely need to be tailored according to tissue compartment, treatment history, cancer status, and cardiovascular risk profile. In addition, the lack of universally validated senescence biomarkers complicates both *in vivo* assessment of senescent cell burden and evaluation of therapeutic response (63, 168). Integration with imaging-based surveillance, functional assessment, multi-omics profiling, and digital monitoring may help overcome some of these limitations, but prospective validation remains essential.

Importantly, senescence-targeting approaches should not be viewed as replacements for established cardio-oncology strategies, such as baseline cardiovascular risk assessment, lifestyle interventions, cardioprotective drugs, and imaging-based surveillance. Instead, they may eventually complement current approaches by targeting a shared biological mechanism linking anticancer therapy, accelerated aging, chronic inflammation, immune–vascular dysfunction and delayed cardiovascular dysfunction. Overall, targeting TIS may provide a novel framework through which cancer therapy, cardiovascular disease, and accelerated biological aging can be addressed within an integrated cardio-oncology perspective. Although substantial biological and translational challenges remain, ongoing advances in senescence biology, biomarker development, and precision therapeutic strategies may ultimately support the development of more personalized approaches aimed at preserving both oncologic efficacy and long-term cardiovascular health in cancer survivors.

## 7 Conclusion

TIS should be considered not merely a byproduct of anticancer therapy, but rather a complex, dynamic, and context-dependent biological process capable of simultaneously influencing tumor behavior, cardiovascular remodeling, immune dysfunction, and accelerated biological aging. In BC survivorship, persistent senescence-associated signaling may therefore represent a candidate, non-exclusive mechanistic link between anticancer treatment, systemic tissue remodeling, and long-term cardiovascular vulnerability.

By integrating molecular senescence pathways with tumor biology, cardiovascular dysfunction, immune remodeling, and aging-related processes, this review positions TIS as a potential contributor at the intersection of oncology and cardiovascular medicine. This perspective may broaden interpretation of cancer therapy-related cardiovascular disease, complementing established models of direct treatment toxicity with the emerging contribution of chronic senescence-associated tissue remodeling and systemic biological aging.

Future advances in biomarker-guided stratification, longitudinal cardiovascular phenotyping, and precision cardio-oncology approaches — including integration of digital health technologies and AI-assisted risk prediction models — may improve the identification of vulnerable patients and facilitate personalized strategies aimed at optimizing both cancer outcomes and long-term cardiovascular health.
